# Role of the Tau N-terminal region in microtubule stabilization revealed by new endogenous truncated forms

**DOI:** 10.1038/srep09659

**Published:** 2015-05-14

**Authors:** Maxime Derisbourg, Coline Leghay, Giovanni Chiappetta, Francisco-Jose Fernandez-Gomez, Cyril Laurent, Dominique Demeyer, Sébastien Carrier, Valérie Buée-Scherrer, David Blum, Joëlle Vinh, Nicolas Sergeant, Yann Verdier, Luc Buée, Malika Hamdane

**Affiliations:** 1 Inserm, UMR-S 1172, Alzheimer & Tauopathies, Jean-Pierre Aubert Research Centre, Faculté de Médecine, IMPRT, F-59045, Lille, France; 2 Université de Lille, F-59045, Lille, France; 3 CHRU-Lille, F-59037, Lille, France; 4 ESPCI Biological Mass Spectrometry and Proteomics USR 3149 CNRS/ESPCI ParisTech, Paris, France

## Abstract

Tau is a central player in Alzheimer's disease (AD) and related Tauopathies, where it is found as aggregates in degenerating neurons. Abnormal post-translational modifications, such as truncation, are likely involved in the pathological process. A major step forward in understanding the role of Tau truncation would be to identify the precise cleavage sites of the several truncated Tau fragments that are observed until now in AD brains, especially those truncated at the N-terminus, which are less characterized than those truncated at the C-terminus. Here, we optimized a proteomics approach and succeeded in identifying a number of new N-terminally truncated Tau species from the human brain. We initiated cell-based functional studies by analyzing the biochemical characteristics of two N-terminally truncated Tau species starting at residues Met11 and Gln124 respectively. Our results show, interestingly, that the Gln124-Tau fragment displays a stronger ability to bind and stabilize microtubules, suggesting that the Tau N-terminal domain could play a direct role in the regulation of microtubule stabilization. Future studies based on our new N-terminally truncated-Tau species should improve our knowledge of the role of truncation in Tau biology as well as in the AD pathological process.

Tau is a microtubule-associated protein (MAP) mainly found in neurons and expressed in the adult human brain as 6 isoforms (ranging from 352 to 441 amino acid residues in length), which are derived from a single gene, *MAPT*, by the alternative splicing of exons 2, 3 and 10^1^. Tau is composed of an amino terminal acidic domain followed by two proline-rich domains and a microtubule-binding domain^2^. The latter contains 3 or 4 microtubule-binding repeats, depending on whether the sequence encoded by exon 10 is included or not^3^. Tau is primarily involved in the regulation of microtubule stability and dynamics as well as axonal transport^4^,^5^. Besides its role as a MAP, Tau exhibits other cellular localizations and functions that have been less investigated^6^,^7^,^8^. Tau proteins aggregate into filaments in a large group of neurodegenerative disorders referred to as Tauopathies, such as Alzheimer's Disease (AD) and Frontotemporal Dementia with Parkinsonism linked to chromosome 17 (FTDP-17)^9^. AD is the most common Tauopathy and form of dementia. One of its neuropathological hallmarks is neurofibrillary degeneration (NFD), characterized by aggregated Tau proteins. Studies have shown that the progression of NFD in cortical brain areas is closely correlated to cognitive impairment in AD, supporting a central role for Tau in AD pathology^10^,^11^. As of now, the mechanisms leading to NFD and its progression are far from being elucidated. Nevertheless, the deregulation of Tau phosphorylation is a key event in the pathological process. Numerous studies suggest that abnormal phosphorylation impedes Tau binding to microtubules, leading on the one hand to the depolymerization and loss of the latter, and on the other hand to the formation of toxic aggregated Tau species^12^. Truncation is another post-translational modification that could have an etiological role in Tau pathology. Numerous cell-based assays show that the truncation of either the C-terminal part of Tau or both the N- and C-terminal parts impacts its biochemical and functional properties and triggers a gain of toxic function^13^,^14^,^15^,^16^. Moreover, animal models based on the expression of particular truncated Tau species are able to reproduce Tau pathology^16^,^17^,^18^. The analysis of AD brains by western blotting (WB) and epitope mapping suggests the occurrence of cleavage sites in both the N-terminal and C-terminal parts of Tau proteins^19^,^20^. While several N- and C-terminally truncated Tau species are observed in AD brains, only a limited number of specific Tau cleavage sites, after residues Asp13, Asp25, Asn368, Glu391 and Asp421, have been identified so far *in situ*. The species generated by these cleavages are found in neurofibrillary tangles, and their occurrence is correlated with the severity of the disease^16^,^21^,^22^,^23^,^24^,^25^,^26^. The precise identification of new Tau cleavage sites is a mandatory step in the generation of appropriate experimental tools with which to investigate their impact on Tau function/dysfunction and obtain new insights into the role of Tau truncation in pathological process but also in physiological framework. In this context, we undertook a challenging proteomics approach to precisely identify new Tau cleavage sites, especially those at the N-terminus, which are less well characterized than those at the C-terminus. We have identified several new N-terminally truncated Tau species in the human brain, with N-terminal residues located throughout the Tau sequence, leading us to expect that these Tau species would be of crucial functional and/or pathological relevance. We therefore initiated cell-based functional studies by analyzing the biochemical characteristics of two N-terminally truncated Tau species. Our results suggest, surprisingly, that the Tau N-terminus could play a direct role in the regulation of microtubule dynamics.

## Results

### Identification of new N-terminally truncated Tau species in the human brain

**Figure 1:**
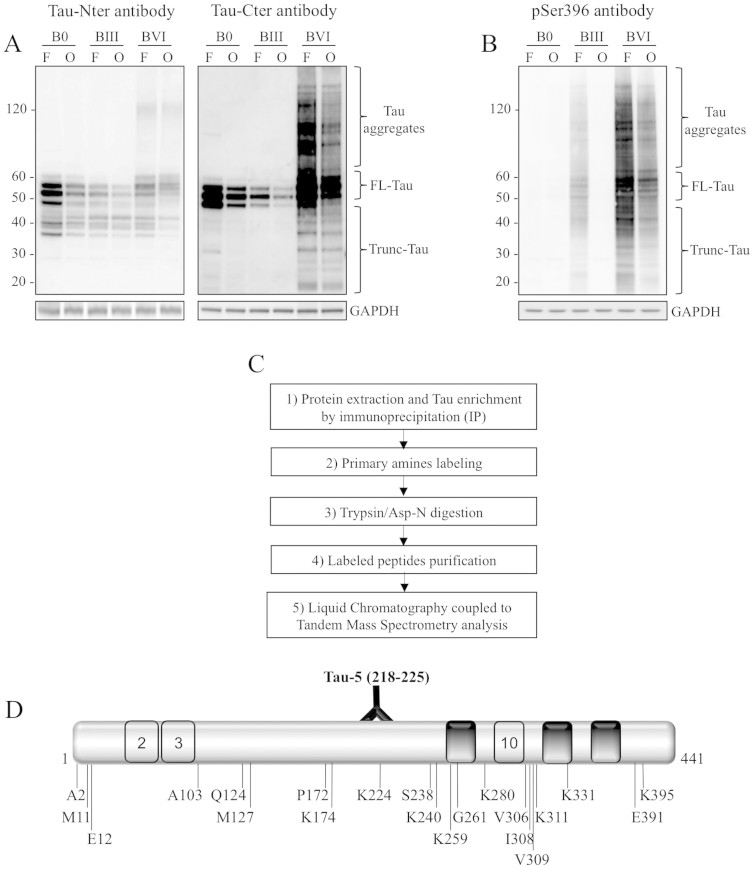
Identification of 21 N-terminal truncation sites of Tau protein from human brain tissue using LC-MS/MS. (A): Characterization of human brain tissue by WB using antibodies directed against the Tau C-terminal (Tau-Cter) and N-terminal (Tau-Nter) ends; representative analysis of tissue from the frontal (F) and occipital (O) cortex of Braak 0 (B0), Braak III (BIII) and Braak VI (BVI) patients. GAPDH was used as a loading control. (B): Characterization of the same human brain tissue by WB using pSer396 antibody. The gels displayed in A and B have been run under the same experimental conditions. Cropped blots are displayed; Full-length blots are presented in supplementary data (as Fig. S7A and Fig. S7B respectively). (C): Proteomics approach developed to identify N-terminal sites of Tau protein; Tau species were immuno-enriched from the human occipital and frontal cortex, labeled with covalently-linked biotin, digested either with trypsin or with Asp-N and analyzed by LC-MS/MS. (D): Representation of the position of identified cleavage sites as well as of the Tau-5 antibody epitope on a schematic Tau sequence (numbering according to the longest Tau isoform).

WB analyses of human cortical brain samples using antibodies directed against the N- and C-terminal parts of Tau protein revealed several truncated Tau species (Fig. 1A), in agreement with previous reports^19^,^20^. These Tau species are also showed by using pSer396 antibody (Fig. 1B). It is worth noting that, as expected, differential phosphorylation of the various Tau species was observed between the samples and between the different brain areas with respect to Braak stages (7). In order to pinpoint new N-terminal truncation sites of Tau, we optimized a proteomic approach (Fig. 1C) in brain samples. The whole range of Tau species was immunoprecipitated (IP) from protein extracts with the Tau-5 antibody, which recognizes amino acid residues 218–225^27^. As shown by WB analysis (Fig. S1), Tau-5 IP allows the purification of full-length Tau (FL-Tau) as well as N- and C-terminally truncated Tau and aggregated species, which display lower and higher molecular weights, respectively. In order to improve detection sensitivity, Tau-5 IP products were then subjected to primary amine labeling using a covalently-linked biotin prior to enzymatic digestion with either trypsin or the endoproteinase Asp-N. Labeled peptides were then purified on streptavidin columns and identified using liquid chromatography-mass spectrometry (LC-MS/MS). This approach resulted in the identification of a number of Tau peptides (Table S1; data compiled from all samples), including some semi-tryptic/semi-Asp-N peptides. Indeed, 21 peptides displayed non-tryptic/non-Asp-N residues at the N-terminus and constituted our set of candidates for N-truncated Tau species (Table 1). Out of them, only 2 (Lys310, Lys 395) can be explained by the activity of possible chymotrypsin contaminant. It should be noted that not all the peptides were N-terminally labeled, in part because of the post-translational modification of amines. Among these N-terminal sites, Ala2 was observed to be Nα-acetylated, as previously reported by Hasegawa et al., who found this Tau species in both normal and AD brains^28^.

These new N-terminal sites were scattered across the Tau sequence (Fig. 1D), and except for Ala2, have not been described before. The N-terminal sites located C-terminally to the Tau-5 epitope were detected probably because they are found in situ in protein complexes with other Tau species, as suggested by WB analysis showing high-molecular-weight Tau species (Fig. S1). It is worth noting that known Tau binding partners such as Hsc70^29^ were also found in our analysis, indicating that the experimental conditions supported co-IP (Fig. S2).

### The Gln124-Tau fragment shows a different phosphorylation pattern from FL-Tau

**Figure 2:**
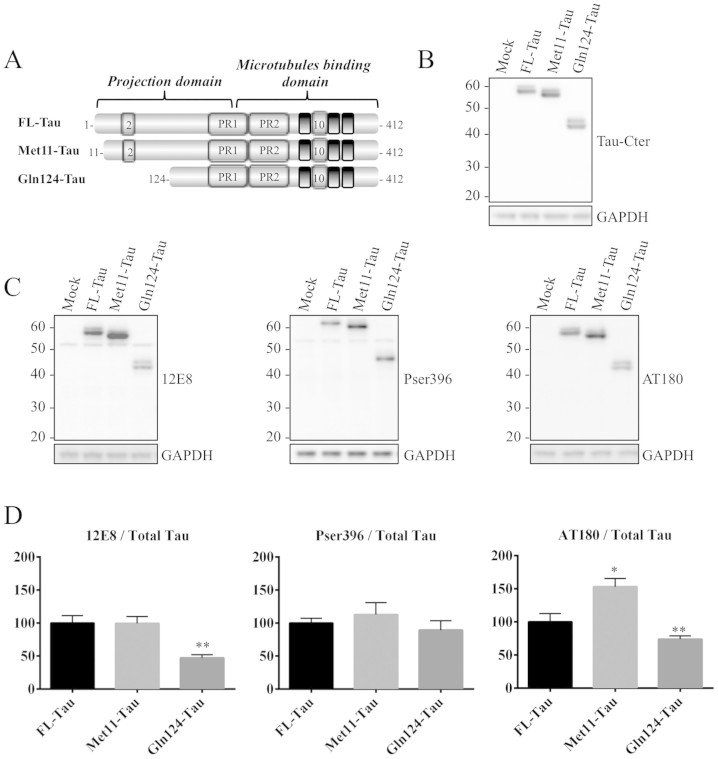
Expression and phosphorylation pattern of truncated Tau proteins. (A): Schematic representation of 1N4R FL-Tau isoform, which includes exons 2 and 10, and the Met11-Tau and Gln124-Tau fragments. PR: proline rich domain. (B): Representative WB analysis using the Tau-Cter antibody of protein extracts from N1E-115 cells transfected with control vector (mock), FL-Tau and the Met11-Tau and Gln124-Tau fragments. GAPDH was used as a loading control. (C-D) Representative WB analysis and densitometric quantifications of phosphorylated epitopes (AT180: pThr231; 12E8: pSer252-pSer356 and pSer396). Quantification was performed by calculating the ratio of phosphorylated Tau to total Tau (Tau-Cter), both relative to GADPH. Error bars indicate SEM. N ≥ 3 independent experiments. *: P ≤ 0.05; **: P ≤ 0.01. Differences between mean values were determined using One-way ANOVA followed by Fisher's LSD post hoc test. The gels displayed in B and C has been run under the same experimental conditions. Cropped blots are displayed; Full-length blots are presented in supplementary data (as Fig. S8A and S8B, respectively).

Regarding the functional relevance of the new identified sites, since they are scattered widely across the Tau sequence, one could expect each cleavage site to have a specific impact, whether exclusive or not, on Tau function and biochemical properties. We therefore initiated functional studies by analyzing the biochemical characteristics of Tau species starting at residues Met11 (Fig. S3A) and Gln124 (Fig. S3B). These residues are located in the Tau projection domain and are present in all Tau isoforms (Fig. 1D). We generated expression vectors containing coding sequences (cDNAs) of FL- and truncated-Tau species (Fig. 2A). These constructs were transfected into the N1E-115 neuroblastoma cell line. Forty-eight hours after transfection, WB analysis using an antibody directed against the C-terminal part of Tau showed immunoreactive bands at the expected molecular weights as well as mobility shifts likely related to particular phosphorylated Tau species (Fig. 2B), but we cannot exclude that the shifts are due to other modifications. An evaluation of phosphorylation status using antibodies to phospho-Tau showed differential phosphorylation patterns (Fig. 2C and 2D). Compared to FL-Tau, Met11-Tau displayed an increase in phosphorylation at the Thr231 epitope (detected by the AT180 antibody), although no difference in phosphorylation was observed at Ser396. Interestingly, Gln124-Tau displayed a decrease in phosphorylation at Thr231 and Ser262/356 (detected by the 12E8 antibody), compared to FL-Tau.

### Cells expressing Gln124-Tau display an increase in α-tubulin acetylation and detyrosination

**Figure 3:**
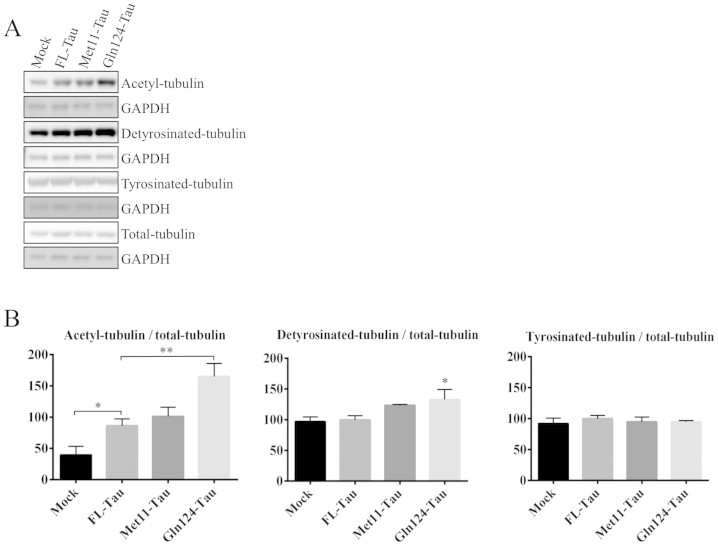
Gln124-Tau increases α-tubulin acetylation and detyrosination. (A): Post-translational modifications of α-tubulin analyzed by WB using protein extracts from N1E-115 cells overexpressing FL-Tau, Met11-Tau or Gln124-Tau. The gels have been run under the same experimental conditions. Cropped blots are displayed; Full-length blots are presented in supplementary data (as Fig. S9). (B): Quantification was performed by calculating the ratio of modified tubulin to total tubulin, both relative to GAPDH. Error bars indicate SEM. N ≥ 5 independent experiments. *: P ≤ 0.05; **: P ≤ 0.01. Differences between mean values were determined using One-way ANOVA followed by Fisher's LSD post hoc test.

Since it has been shown that phosphorylation at Ser262/Ser356 strongly decreases the binding of Tau to microtubules^30^,^31^, we asked whether the truncation of the N-terminal part of Tau could have any consequences for Tau function in microtubule stabilization. To investigate the properties of N-terminally truncated Tau species with regard to microtubules, we first compared the effect of their expression on microtubule modifications, in particular the acetylation of α-tubulin, which is a hallmark of stable microtubules^32^. WB analysis of transiently transfected N1E-115 cells showed that, as expected, cells expressing FL-Tau displayed an increase in the level of α-tubulin acetylation (Fig. 3). Regarding the effect of N-terminally truncated Tau, the Met11-Tau fragment showed the same properties as FL-Tau while Gln124-Tau was associated with a markedly increased level of acetylated tubulin compared to FL-Tau. Given that Tau can interact with and inhibit the function of HDAC6, the main tubulin deacetylase^33^, we investigated whether the increase in tubulin acetylation in cells expressing Gln124-Tau was related to a stronger inhibition of HDAC6 activity. We measured HDAC activity in cytoplasmic extracts, since HDAC6 is mainly cytoplasmic compared to other HDAC members. Our data (Fig. S4) show that there was no significant difference in HDAC activity between cells transfected with FL-Tau and those with N-terminally truncated species, indicating that the effect on tubulin acetylation is not related to differences in HDAC6 activity. Instead, this effect could be a consequence of improved microtubule polymerization and/or stabilization. In agreement with this possibility, the investigation of another post-translational modification of tubulin, detyrosination, which is also representative of a stable population of microtubules^34^, revealed that it was significantly increased with Gln124-Tau when compared to FL-Tau (Fig. 3).

### Gln124-Tau binds more strongly to microtubules than FL-Tau and protects against depolymerization

Since tubulin acetylation and detyrosination occur once microtubules are polymerized, the increase in their levels in cells expressing Gln124-Tau is likely a consequence of improved microtubule polymerization and/or stabilization. To investigate the capacity of Tau species to promote microtubule polymerization in cells, we analyzed their ability to induce neurite-like extensions in response to cytochalasin B. The process formation in this experimental test is an indicator of the ability of Tau to assemble and polymerize microtubules. Forty-eight hours after transfection, N1E-115 cells were subjected to cytochalasin B treatment for 1 h. In agreement with well-established reports^35^, immunolabeling analysis by confocal microscopy showed that the breakdown of the cortical actin network by cytochalasin B allowed cells expressing FL-Tau to lengthen microtubule bundles into cellular extensions that looked like neurites (Fig. S5A). An analysis of cells expressing N-terminally truncated species showed no evident difference in the number of cells displaying cellular extensions compared to cells expressing FL-Tau (Fig. S5B). It is worth noting that, in accordance with the reported *in vitro* studies, the ability of Tau to assemble and polymerize microtubules requires the domains involved in microtubule binding and assembly: the second proline-rich domain, the microtubule-binding repeats as well as inter-repeat regions^36^,^37^. Indeed, in our experiments, cells overexpressing a Tau fragment that is truncated in the second proline-rich domain and a Tau fragment that is truncated in the first repeat domain do not display any process formation (Fig. S6).

**Figure 4:**
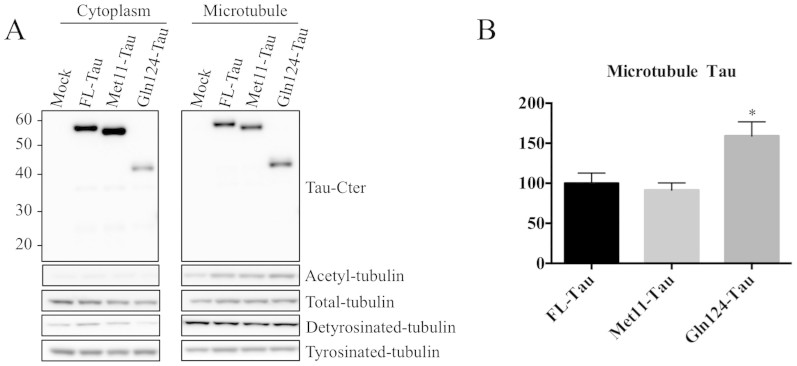
Gln124-Tau binds more efficiently to microtubules than FL-Tau. (A): Representative WB analysis of microtubule fractions from N1E-115 cell extracts transiently transfected with FL-Tau, Met11-Tau or Gln124-Tau fragments. The purity of the fractions was evaluated using an antibody to acetylated α-tubulin. The gels have been run under the same experimental conditions. Cropped blots are displayed; Full-length blots are presented in supplementary data (as Fig. S10). (B): Quantification was performed by calculating the ratio of microtubule-associated Tau to total Tau. Error bars indicate SEM. N ≥ 3 independent experiments. *: P ≤ 0.05. Differences between mean values were determined using One-way ANOVA followed by Fisher's LSD post hoc test.

Next, we examined whether there was any difference in microtubule stabilization. We first compared the microtubule-association properties of FL-, Met11- and Gln124-Tau species. Transiently transfected cells were fractionated into a cytosol fraction (containing free Tau) and a microtubule fraction (containing microtubule-associated Tau), and were analyzed by WB (Fig. 4A). As expected, both acetylated and detyrosinated tubulin forms were mainly present in the microtubule-enriched fraction. Immunolabeling with a Tau antibody revealed that all Tau species were found in both microtubule and cytosol fractions. Nevertheless, the proportion of Gln124-Tau was higher in the microtubule-enriched fraction (Fig. 4B), suggesting that this fragment binds more strongly to microtubules compared to FL-Tau or Met11-Tau.

**Figure 5:**
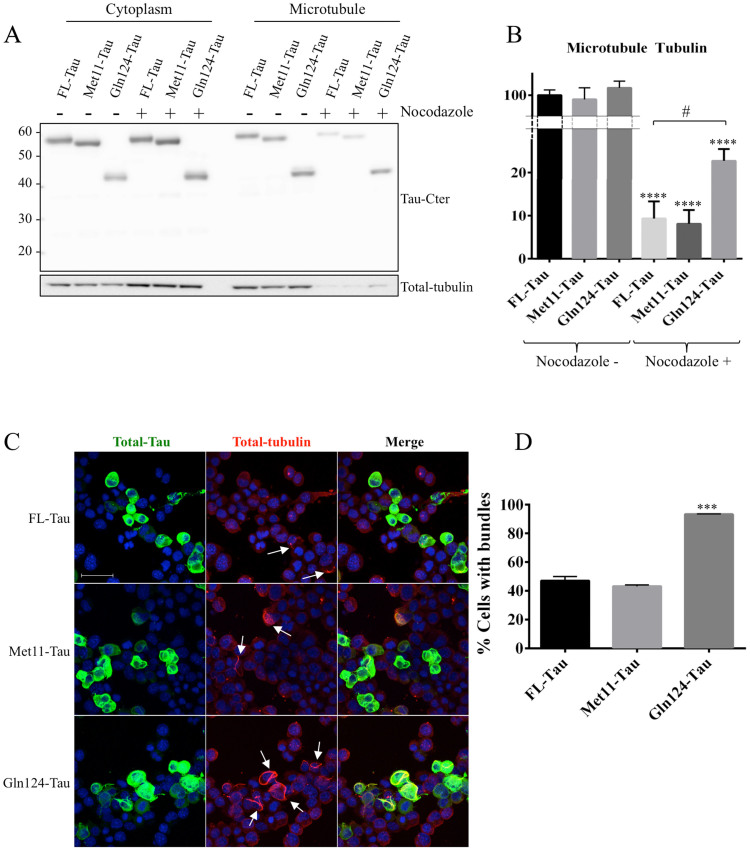
Gln124 protects cells more effectively against microtubule depolymerization than FL-Tau. (A): Representative analysis of Tau species and total tubulin distribution in microtubule fractions, performed after 20 minutes of nocodazole treatment. (B): Quantification was performed by calculating the ratio of tubuline present in the microtubule fraction to total tubuline. The Cropped blots are displayed; Full-length blots are presented in supplementary data (as Fig. S11). (C): Confocal imaging of N15-115 cells transfected with Tau and the truncated species Met11-Tau and Gln124-Tau, and treated with nocodazole. Cells harbor bundles are designated by white arrows. Scale bar: 50 μM. (D): Quantification of cells expressing Tau, Met11-Tau or Gln124-Tau which display bundles after 20 minutes of nocodazole treatment. Error bars indicate SEM. N ≥ 3 independent experiments. ***: P ≤ 0.001. Differences between mean values were determined using One-way ANOVA followed by Fisher's LSD post hoc test.

We then tested the ability of Gln124-Tau to protect microtubules from depolymerization induced by nocodazole treatment. The nocodazole-resistance experiment allows the capacity of Tau to protect microtubules from depolymerization once they are assembled to be evaluated, and is hence an indicator of microtubule stability and dynamics. Transfected N1E-115 cells were treated for 20 minutes with nocodazole and subjected to cytosol/microtubule fractionation for WB analysis of microtubule depolymerization and Tau release from microtubules. Nocodazole treatment induced microtubule depolymerization and Tau release from microtubules, as shown by decreased tubulin and Tau immunolabeling, respectively, in the microtubule fraction from treated cells (Fig. 5A). However, cells expressing Gln124-Tau were markedly more resistant to nocodazole treatment compared to cells expressing FL-Tau. Indeed, higher levels of tubulin were observed in the microtubule fraction from nocodazole-treated Gln124-Tau cells compared to cells transfected with the other Tau species (Fig. 5A and 5B). To confirm these data, transfected N1E-115 cells were treated for 20 minutes with nocodazole and the disruption of Tau-induced microtubule bundles analyzed by confocal microscopy (Fig. 5C). The results show that unlike cells expressing FL-Tau or Met11-Tau, microtubule bundles were still present in almost all cells expressing Gln124-Tau after 20 min of treatment (Fig. 5C and 5D). Hence, microtubules in cells expressing Gln124-Tau are less sensitive to depolymerization than microtubules in cells expressing FL-Tau or Met11-Tau.

## Discussion

In this work, using a dedicated proteomics approach, we identified new N-terminally truncated Tau species in the human brain by identifying their precise primary amine residues. Our finding lays the groundwork for the development of appropriate tools with which to monitor species truncated at these newly identified sites under physiological and pathological conditions, and obtain new insights into the role of truncation in Tau function and dysfunction.

Truncation is one of the post-translational modifications of Tau encountered in AD brains, as shown by the detection of several Tau species truncated at the N- and C-terminus^19^,^20^. The determination of the precise cleavage sites of some of these Tau species was initially made possible by epitope mapping^21^,^22^,^23^,^24^,^25^. While the main C-terminal cleavage sites (Glu391 and Asp421) are well characterized, among the plethora of N-terminally truncated species, only two have been identified in situ^24^,^25^. The purpose of our proteomics approach was to simplify peptide mixtures before MS/MS identification, in order to enhance the probability of identifying the N-terminal extremities of these Tau species. Our approach required the labeling of available amino groups on intact proteins using an N-hydroxysuccinide (NHS) ester derivative of biotin. Biotinylated peptides were then purified using streptavidin and analyzed by MS/MS. Indeed, as we started with human brain tissue, we needed to avoid multiple peptide-purification steps in order to strike a balance between peptide loss and N-terminal enrichment, although effective protocols have been published^38^. In order for a peptide to be validated as a candidate for N-terminal Tau truncation, its amino-terminal extremity should not be a cleavage site for the protease used (trypsin or Asp-N) and it should be found in several samples in order to reduce the probability of random cleavage by the enzyme, even if trypsin has been reported to be very specific^39^. It should be noted that labeling of the amino-terminal group was not considered an absolute validation criterion due to the fact that our labeling procedure was probably not complete under the experimental conditions used. For some peptides, the amino-terminal group was blocked by post-translational modifications, such as the Nα-acetylation of Ala2. Here, we identified a number of new Tau N-terminal sites, but there is a gap between the detection and the quantification of these Tau species in normal and AD brains. Right now, we cannot say with any certainty which cleavage sites are relevant to the disease process and which are specific to the physiological context. Likewise, the impact of post-mortem delay cannot be unequivocally addressed. The present approach allows for the identification but not the quantification of the amino-truncated species. In fact, to obtain reliable quantitative results, it is necessary to avoid the IP and N-terminal labeling of Tau proteins we used in our study. To reach this goal, the method of choice would be the development of specific antibodies, as was initially done for C-terminally truncated Tau species, especially those ending at Glu391 and Asp421^21^,^22^,^23^. Hence, our interesting findings provide the basis for the development of appropriate tools with which to monitor truncated species that start at these newly identified sites, as well as the corresponding C-terminally truncated species, under physiological and pathological conditions. Moreover, these tools would be of interest in the diagnostic field.

Regarding the consequences of truncation to Tau protein properties, our initial cell-based studies show that the ability of Tau to stabilize microtubules is greater when the N-terminal part is truncated. Indeed, the Gln124-Tau fragment showed a stronger ability to bind microtubules and protect them from depolymerization compared to FL-Tau. In agreement with this supposition, cells expressing the Gln124-Tau fragment display a significant increase in post-translational modifications characteristic of stable microtubules (α-tubulin acetylation and detyrosination). It is widely accepted, based mainly on *in vitro* analyses of Tau fragments generated by amino-terminal deletions, that Tau binds microtubules and regulates their stabilization and polymerization through its C-terminal part. These earlier studies indicated that the direct effects of Tau with regard to microtubules involves a region encompassing amino acid residues 215–358, which contains the second proline-rich domain, the microtubule-binding repeats as well as inter-repeat regions^36^,^37^. The role of the Tau amino-terminal domain with regard to microtubules has been reported as being indirect, such as by the regulation of microtubule spacing^40^ and functions^41^,^42^. Nevertheless, in lines with our data, *in vitro* studies of the impact of missense mutations encountered in Tauopathies (mutations at the Arg5 and at Gly55 residues) suggest that the modification of the amino-terminal domain of Tau could directly impact microtubules^43^,^44^,^45^. Besides, a recent *in vitro* attempt to improve mechanisms of Tau interaction with microtubules based on the use of Tau fragments generated by limited proteolysis has shown that the Tau fragment Ser208-Ser324 binds more tightly to microtubules than FL-Tau and favors their assembly^46^. In agreement with these *in vitro* assays, our cell-based study of the N-terminally truncated Tau fragment (Gln124-Tau) newly identified *in situ* suggests that the amino-terminal domain of Tau could directly regulate its binding and stabilization of microtubules. To further characterize the Gln124-Tau fragment, it would be of interest to evaluate, on the one hand, whether the observed effects are isoform-dependent, and on the other hand, the impact of Gln124-Tau on the functions of FL-Tau. Indeed, the current work was performed in a cell line that does not display detectable levels of endogenous FL-Tau.

Regarding the mechanisms underlying the gain of function displayed by the Gln124-Tau fragment, one explanation could be related to the fact that the Tau protein is prone to adopt a “paperclip” conformation as a result of intra-molecular interactions between the N-terminal and C-terminal domains^47^,^48^. Hence, N-terminal truncation would be expected to unfold Tau from this conformation and to expose the microtubule-binding domain. This explanation is unlikely under our experimental conditions, since we find no obvious difference with regard to microtubule stabilization between the Met11-Tau fragment and FL-Tau. A more plausible explanation would be that Gln124-Tau, due to the truncation of the negatively charged N-terminus, displays enhanced binding to the negative surface of microtubules.

Concerning the biological significance of this gain of function, sustained microtubule stabilization is likely to have a deleterious effect on neurons by impairing synaptic plasticity and microtubule-dependent transport. Indeed, mutations in FTDP-17 that lead to an increase in 4R Tau isoforms, which stabilize microtubules more strongly than 3R isoforms, are the cause of neuronal death and dementia^49^. Moreover, given that the microtubule-severing proteins katanin and spastin have a more potent effect on stable microtubules^50^,^51^, a sustained increase in microtubule stability and its associated α-tubulin acetylation and detyrosination might lead to the increased recruitment of severing enzymes and microtubule loss due to excess cutting^52^,^53^.

In summary, this work identifies new N-terminally truncated Tau species that occur in the human brain and that have potential relevance to Tau biology and likely to AD-related Tauopathies. Our preliminary cell-based studies suggest, interestingly, that the N-terminal part of Tau could be directly involved in the regulation of microtubule stabilization. N-terminal truncation may also influence other Tau properties such as polymerization^22^ and cellular localization^54^. Future investigations based on our newly identified N-terminally truncated Tau species, in particular Gln124-Tau, should improve our knowledge as to the role of truncation in Tau biology as well as in the AD pathological process.

## Methods

### Human tissue samples

Human brain autopsy samples were from the Lille NeuroBank collection (Centre de Ressources Biologiques du CHRU de Lille). Informed consent was obtained from all subjects. The Lille NeuroBank has been declared to the French Research Ministry by the Lille Regional Hospital (CHRU-Lille) on August 14, 2008 under the reference DC-2000-642 and fulfils the criteria defined by French Law regarding biological resources, including informed consent, ethics review committee approval and data protection. The study was approved by the ethics review committee of Lille NeuroBank. The Table 2 indicates the stage of Tau pathology, categorized on the basis of neuropathological characteristics according to Braak and Braak^11^.

### Cell culture and transfection

N1E-115 mouse neuroblastoma cells were grown in Dulbecco's modified Eagle's medium supplemented with 10% fetal calf serum without pyruvate, 2 mM L-glutamine and 50 units/ml penicillin/streptomycin (Invitrogen) in a 5% CO_2_ humidified incubator at 37°C.

Transfection with plasmid constructs was performed 24 hours after cell seeding into six-well plates (for biochemical studies) or on culture slides (for immunocytochemistry), using ExGen500 (Euromedex) according to the manufacturer's instructions, for 48 hours.

### Plasmid constructs

Expression vectors carrying cDNA for FL and N-terminally truncated Tau 1N4R isoform were generated using the In-Fusion cloning Kit (Clontech), and PCR primers were designed to clone inserts into the EcoRI site of pcDNA3.1 (Invitrogen). Each cDNA fragment was amplified by PCR (DyNAzymeTM EXT DNA polymerase, New England BioLabs) from pcDNA3.1-Tau4R^55^. Forward primers were designed to contain the Kozak consensus sequence and are as follows:

For **FL-Tau**, 5′-CAGTGTGGTGGAATTCGCCACC**ATG**GCTGAGCCCCGCCAGGAGTT-3′;

**For Met11-Tau**, 5′-CAGTGTGGTGGAATTCGCCACC**ATG**GAAGATCACGCTGGGACGT-3′;

**For Gln124-Tau**, 5′-CAGTGTGGTGGAATTCGCCACC**ATG**CAAGCTCGCATGGTCAGTAA AAGCAAAGACGGG-3′;

**For Val229-Tau**, 5′-CAGTGTGGTGGAATTCGCCACCATGGTCCGTACTCCACCCAAGTCGCCGTCT-3′;

**For Gly261-Tau**, 5′-CAGTGTGGTGGAATTCGCCACCATGGGCTCCACTGAGAACCTGAAGCACCAGC-3′.

### The reverse primer for all amplifications was:

5′-GATATCTGCAGAATTCTCACAAACCCTGCTTGGCCAGGG AGGCA-3′.

DNA sequencing was carried out for construct validation.

### Protein extractions

Tissues were homogenized by sonication in a buffer containing 0.32 M sucrose and 10 mM Tris-HCl, pH 7.4. For biochemical characterization and immunoprecipitation, samples were prepared using IP-buffer: 100 mM NaCl, 110 mM KOAc, 0.5% Triton X-100, Buffering Salts pH 7.4 (Invitrogen) with protease inhibitors (Complete w/o EDTA, Roche), sonicated and centrifuged at 2500 × g for 5 min.

For cell protein extracts; cells were washed using PBS and harvested in ice-cold RIPA buffer: 150 mM NaCl, 1% NP40, 0.5% sodium deoxycholate, 0.1% SDS, 50 mM Tris-HCl, pH 8.0, completed with protease inhibitors. Protein concentrations were determined using the BCA Assay Kit (Pierce).

### Immunoprecipitation (IP)

IP was performed using the Dynabeads Co-Immunoprecipitation Kit 143.21D (Invitrogen). Briefly, the first step consisted of coupling 25 mg of magnetic beads with 250 μL of Tau-5 antibody (Invitrogen) according to the manufacturer's protocol. Then, 1250 μg of protein extract were added to the bead-antibody complex and incubated overnight at 4°C. After washing with IP buffer, IP products were obtained by adding the elution buffer: buffering salts pH 2.8 (Invitrogen). A small aliquot of this fraction was analyzed using WB and the rest lyophilized using a speed-vac for proteomics analysis.

### Identification of peptides by capillary liquid chromatography-tandem mass spectrometry (LC-MS/MS)

IP Tau solubilized in PBS (pH 8.0) was reduced and alkylated (DTT, 10 mM final, 2 h, 37°C; iodoacetamide, 50 mM final, 30 min in the dark). After 3 washes with PBS on a microcolumn (Millipore, cut off 10 kDa), primary amines were labeled with NHS-biotin (Thermo scientific) according to the manufacturer's instructions (1 mM, 2 h, RT). Then, NHS was saturated using 200 mM hydroxyalamine, and proteins washed 3 times with ammonium bicarbonate (50 mM). The protein extract was then divided in two parts, one for proteolysis using trypsin (10 ng modified sequencing-grade trypsin; Roche; 37°C, overnight) and the other for proteolysis with Asp-N (10 ng, Roche; 37°C, overnight). The resulting peptides were then purified using streptavidin beads (Thermo scientific) according to the manufacturer's recommendations. 300 μL of resins were incubated for 1 h at RT with the samples. After washing with PBS, bound peptides were eluted with DTT (5 mM final, 30 min, RT), acidified with 10 μL of 10% aqueous formic acid and desalted using ZipTip C18 microcolumns (Millipore).

Peptides (2 μL) were purified on a capillary reversed-phase column (nano C18 Acclaim PepMap100 Å, 75 μm i.d., 15 cm length; Dionex) at a constant flow rate of 220 nL/min, with a gradient of 2% to 40% of buffer B in buffer A, for 45 min; buffer A: water/acetonitrile/formic acid 98:2:0.1 (vol/vol/vol); buffer B: water/ACN/formic acid 10:90:0.1 (vol/vol/vol). Coupling with the mass spectrometer was done using a NanoMate (Advion; Voltage: +1.70 kV; Spray Sensing Enabled; Below 10.0 nA or above 2000.0 nA for 5 secs; Chip ID: A8E159AK). The MS analysis was performed on a Fourier transform ion cyclotron resonance (FT-ICR) mass spectrometer (LTQ-FT Ultra; ThermoFisher Scientific) with the top-seven acquisition method: MS resolution 60,000, mass range 470–2,000 Da, followed by MS/MS (LTQ) of the seven most intense peaks, with a dynamic exclusion of 90 s.

The raw data were processed using Xcalibur 2.0.7 software. The database search was done using the Mascot search engine (Matrix Science Mascot 2.2.04) on a homemade protein databank containing the 6 human Tau isoforms and some contaminants, or on the entire SwissProt protein databank. Proteome Discoverer 1.3 (ThermoFisher Scientific) and Mascot were used to search data and filter the results. The following parameters were used: MS tolerance 5 ppm; MS/MS tolerance 0.5 Da; semi-tryptic or semi-Asp-N peptides; one missed cleavage allowed; partial modifications: carbamidomethylation (C), oxidation (M), phosphorylation (ST), acetylation (N-term), thiopropionation (N-ter, K).

The mass spectrometry proteomics data have been deposited to the ProteomeXchange Consortium (http://proteomecentral.proteomexchange.org) via the PRIDE partner repository^56^ with the dataset identifier PXD001353 and DOI 10.6019/PXD001353.

### Western blotting (WB)

Protein extraxts were standardized at 1 μg/μL with LDS 2X supplemented with a reducing agent (Invitrogen) and denatured at 100°C for 10 min. Proteins were then separated with SDS-PAGE using precast 4–12% Bis-Tris NuPage Novex gels (Invitrogen). Proteins were transferred to 0.45 μM nitrocellulose membranes (Amersham^TM^ Hybond ECL), which were saturated with 5% dry non-fat milk in TNT buffer; 140 mM NaCl, 0.5% Tween20, 15 mM Tris, pH7.4, or 5% bovine serum albumin in TNT (Sigma) depending on the primary antibody. Membranes were then incubated with the primary antibodies (Table S2) overnight at 4°C, washed with TNT three times for ten minutes, incubated with the secondary antibodies (Vector) and washed again before development. Immunolabeling was visualized using chemiluminescence kits (ECL^TM^, Amersham Bioscience) on an LAS-3000 acquisition system (Fujifilm). Labeling was quantified with ImageJ software (Scion Software).

### Immunocytochemistry

Cells were plated on culture slides (BD Biosciences) coated with poly-D-lysine (Sigma). Twenty-four hours after plating, cells were transfected according to the protocol previously described. Two days post-transfection, cell were subjected to the appropriate treatment: 20 μM Cytochalasin B (Sigma) for 1 h or 10 μM Nocodazole (Sigma) for 20 min. Cells were then washed with prewarmed PBS, fixed for 20 min with ice-cold methanol, permeabilized with a solution of PBS and 0.2% Triton X-100, washed again with PBS and saturated with PBS with 2% BSA. Cells were then incubated with the primary antibodies overnight at 4°C (Table S2), washed with PBS three times 10 min, incubated with secondary antibodies, and washed. Laser-scanning confocal microscopy was performed using a Zeiss LSM 710 laser scanning system.

### Cell fractionation into cytosolic and microtubule fractions

An equivalent number of transfected cells was recovered in equal volumes of warmed lysis buffer: 1 mM MgCl_2_, 2 mM EGTA, 30% glycerol, 0.1% Triton X-100, 80 mM Pipes, pH 6.8, complemented with protease inhibitors. After ultracentrifugation at 100 000 × g at 21°C for 18 min, supernatants were collected as cytosolic fractions. The remaining pellets were washed and recovered as microtubule fractions by sonication in Ripa buffer (in a volume equal to that of cytosolic fractions). Samples were mixed with LDS buffer, and equal volumes were loaded for SDS-PAGE and analyzed by immunoblotting.

The methods were carried out in “accordance” with the approved guidelines.

## Author Contributions

L.B. and M.H. conceived and managed the project; M.D., L.B. and M.H. designed experiments and analyzed data; M.D. performed most experiments; C.Le. contributed to cell based experiments; G.C. and Y.V. performed LC-MS/MS experiments; F.-J.F.-G. and C.La. contributed to biochemical experiments; D.D. and S.C. contributed to generation and preparation of expression vectors; V.B.-S. supervised processing and biochemical analyses of human brains; D.B. and N.S. contributed to data analyses and manuscript reviewing; G.C., J.V. and Y.V. analyzed LC-MS/MS data and prepared related figures; L.B. and Y.V. contributed to manuscript reviewing; M.D. and M.H. wrote the main manuscript text and prepared the major figures.

## Supplementary Material

Supplementary Information Supplementary Information

## Figures and Tables

**Table 1:** Identification of 21 N-terminal truncation sites of Tau protein by LC-MS/MS. Semi-tryptic and semi-Asp-N peptides detected in several samples are shown, with the corresponding first amino acid residue and N-terminal modifications (numbering of N-terminal residues correspond to the N-terminal cleavage site identified). In bold, identified cleavage sites on the N-terminal side of the Tau-5 epitope

Residue position	Detected peptide	Modification (s)
**2**	**AEPRQEFEVME**	**N-Term(Acetyl)**
**2**	**AEPRQEFEVME**	**N-Term(Acetyl); M10(Oxidation)**
**11**	**MEDHAGTYGLGDR**	**N-Term(Thio-)**
**11**	**MEDHAGTYGLGDR**	**N-Term(Thio-); M1(Oxidation)**
**12**	**EDHAGTYGLGDR**	**N-Term(Thio-)**
**103**	**AEEAGIGDTPSLEDEAAGHVTQAR**	
**124**	**QARMVSKSK**	**K7(Thio-); K9(Thio-)**
**124**	**QARMVSKSK**	**K7(Thio-)**
**124**	**QARMVSKSK**	**M4(Oxidation); K7(Thio-); K9(Thio-)**
**124**	**QARMVSKSKDGTGS**	**K7(Thio-); K9(Thio-); 1 miss cleavage**
**127**	**MVSKSKDGTGS**	**K4(Thio-); K6(Thio-); 1 miss cleavage**
**172**	**PAKTPPAPK**	**K3(Thio-), 1 miss cleavage**
**174**	**KTPPAPKTPPSSGEPPK**	**N-Term(Thio-); K7(Thio-), 2 misses cleavages**
**174**	**KTPPAPKTPPSSGEPPKSG**	**K1(Thio-)**
224	KKVAVVR	N-Term(Thio-); K2(Thio-); 2 misses cleavages
238	SAKSRLQTAPVPMP	N-Term(Thio-)
238	SAKSRLQTAPVPMP	K3(Thio-); M13(Oxidation)
240	KSRLQTAPVPMP	N-Term(Thio-)
240	KSRLQTAPVPMP	N-Term(Thio-); M11(Oxidation)
259	KIGSTENLK	K1(Thio-); 1 miss cleavage
261	GSTENLKHQPGGGK	K7(Thio-); 1 miss cleavage
280	KKLDLSNVQSK	N-Term(Thio-); K2(Thio-); 2 misses cleavages
306	VQIVYKPVDLSK	K6(Thio-)
306	VQIVYKPV	
308	IVYKPVDLSK	N-Term(Thio-); K4(Thio-)
308	IVYKPVDLSK	K4(Thio-)
309	VYKPVDLSK	K3(Thio-)
311	KPVDLSK	N-Term(Thio-); K7(Thio-)
311	KPVDLSKVTSK	K1(Thio-); K7(Thio-); 1 miss cleavage
311	KPVDLSKVTSK	K7(Thio-); 1 miss cleavage
331	KPGGGQVEVK	N-Term(Thio-)
391	EIVYKSPVVSG	K5(Thio-)
391	EIVYKSPVVSG	
395	KSPVVSGDTSPR	N-Term(Thio-); 1 miss cleavage

**Table 2:** Summary of brain tissues used for LC-MS/MS analysis

Sample number	Braak Stages	Tissue	Age	PMD (H)
1	Braak 0	Frontal	22	24
2		Occipital		
3	Braak 0	Occipital	26	Unknown
4				
5	Braak III	Frontal	82	48
6		Occipital		
7	Braak III	Frontal	76	10
8		Occipital		
9		Parietal		
10		Temporal		
11	Braak VI	Frontal	56	26
12		Occipital		
