# Supplementary material for: Role of the Tau N-terminal region in microtubule stabilization revealed by new endogenous truncated forms
Source: Sci Rep. 2015 May 14;5:9659. doi: 10.1038/srep09659 (PMC4431475; doi:10.1038/srep09659)
Supplement: Supplementary Information [file srep09659-s1.pdf]

Title:

Role of the Tau N-terminal region in microtubule stabilization revealed by new endogenous truncated forms

Authors: Maxime Derisbourg[1,2,3], Coline Leghay[1,2,3], Giovanni Chiappetta[4], Francisco-Jose Fernandez-Gomez[1,2], Cyril Laurent[1,2], Dominique Demeyer[1,2,3], Sébastien Carrier,[1,2,3], Valérie Buée-Scherrer[1,2,3], David Blum[1,2,3], Joëlle Vinh[4], Nicolas Sergeant[1,2,3], Yann Verdier[4], Luc Buée[1,2,3], Malika Hamdane*[1,2,3].

Figure S1: Representative WB of human frontal cortex from patients displaying Braak 0 (BO) and Braak VI (BVI) neuropathology after immunoprecipitation of Tau proteins with the Tau-5 antibody. Unb: unbound, Tau5-IP: eluted proteins, Beads: non eluted proteins. The gels have been run under the same experimental conditions. Cropped blots are displayed; Full-length blots are presented in these supplementary data (as Fig. S12)

Figure S2: Validation of co-IP conditions. A: Sequence coverage of the Hsc70 protein (P11142) co-purified with the Tau protein. B: MS/MS spectra of a unique peptide of the Hsc70 protein.

Figure S3: MS/MS spectra of: A. the N-terminally labeled peptide M(thio-propionyl)EDHAGTYGLGD; B: the QARMVSK(thio-propionyl)SK peptide.

Figure S4: Differences in α-tubulin acetylation are not related to differences in HDAC6 expression or activity. A: Nuclear and cytoplasmic fractions of N15-115 cells expressing FL-Tau, Met11-Tau and Gln124-Tau were analyzed by WB to investigate the level of HDAC6. The gels have been run under the same experimental conditions. Cropped blots are displayed; Full-length blots are presented in these supplementary data (as Fig. S13). B: Quantification of the HDAC6/actin ratio in the cytoplasmic fraction. C: Measurements of HDAC6 activity on cytoplasmic extracts. Tubacin was used as a positive control of HDAC6 inhibition. Error bars indicate SEM. $N \geq 3$ independent experiments **: $P \leq 0.01$. Differences between mean values were determined using One-way ANOVA followed by Fisher's LSD post hoc test.

Figure S5: Effect of FL-Tau, Met11-Tau and Gln124-Tau fragments on neuritic like extension induced by cytochalasin B . A: Confocal imaging of N15-115 cells overexpressing Tau (green) and treated with cytochalasin B for 1 hour. Neuritic extension can be observed following tubulin labeling (red). Scale bar: 50 µM. B: Quantification of cells expressing Tau species which display neurites extension after cyto treatment. NT: non treated; cyto: Cytochalasin B

Figure S6: Expression pattern of Val229-Tau and Gly261-Tau fragments and effect on neuritic like extension and microtubular distribution, compared to FL-Tau, Met11-Tau and Gln124-Tau fragments. A: Schematic representation of 1N4R FL-Tau isoform, which includes exons 2 and 10, and the Met11-Tau, Gln124-Tau, Val229-Tau and Gly261-Tau fragments. PR: proline rich domain; Representative WB analysis using the Tau-Cter antibody of protein extracts from N1E-115 cells transfected with control vector (mock), FL-Tau and the fragments Met11-Tau, Gln124-Tau, Val229-Tau and Gly261-Tau. GAPDH was used as a loading control. Cropped blot is displayed. **B:** Effect of Val229-Tau and Gly261-Tau fragments, compared to FL-Tau, Met11-Tau and Gln124-Tau

fragments on neuritic like extension induced by cytochalasin B; Confocal imaging of N15-115 cells overexpressing Tau (green) and treated with cytochalasin B for 1 hour. Neuritic extension can be observed following tubulin labeling (red). Scale bar: 50 μM; Histograms represent quantification of cells expressing Tau species, which display neuritis extension after cyto treatment. NT: non treated; cyto: Cytochalasin B. **C:** Representative WB analysis of microtubule fractions from N1E-115 cell extracts transiently transfected with FL-Tau and Tau fragments: Met11-Tau, Gln124-Tau, Val229-Tau and Gly261-Tau. The purity of the fractions was evaluated using an antibody to acetylated α-tubulin. Cropped blot is displayed; Quantification was performed by calculating the ratio of microtubule-associated Tau to total Tau. Error bars indicate SEM. N ≥ 3 independent experiments. *: $P \leq 0.05$, **: $P \leq 0.01$. Differences between mean values were determined using One-way ANOVA followed by Fisher's LSD post hoc test.

Figure S7: Full-length blots of cropped blots presented in Fig. 1 of the main paper. A: related to Fig.1A of the main paper. B: related to Fig.1B of the main paper.

Figure S8: Full-length blots of cropped blots presented in Fig. 2 of the main paper. A: related to Fig.2B of the main paper. B: related to Fig.2C of the main paper.

Figure S9: Full-length blots of cropped blot presented in Fig. 3A of the main paper.

Figure S10: Full-length blots of cropped blot presented in Fig. 4A of the main paper.

Figure S11: Full-length blots of cropped blot presented in Fig. 5A of the main paper.

Figure S12: Full-length blots of cropped blot presented in Fig. S1 of these supplementary informations.

Figure S13: Full-length blots of cropped blot presented in Fig. S4 of these supplementary informations.

Table. S1A

Full list of semi-tryptic and semi-Asp-N peptides

Residue position	N-ter cleavage	Detected peptide	Modification (s)	MH+ Da
2	unspecific	AEPRQEFEVME	N-Term(Acetyl)	1406,62499
2	unspecific	AEPRQEFEVME	N-Term(Acetyl); M10(Oxidation)	1422,62026
11	unspecific	MEDHAGTYGLGDR	N-Term(Thio-)	1509,60953
11	unspecific	MEDHAGTYGLGDR	N-Term(Thio-); M1(Oxidation)	1525,60238
12	unspecific	EDHAGTYGLGDR	N-Term(Thio-)	1378,56938
103	unspecific	AEEAGIGDTPSLEDEAAGHVTQAR		2424,12487
124	unspecific	QARMVSKSK	K7(Thio-); K9(Thio-)	1210,57489
124	unspecific	QARMVSKSK	K7(Thio-)	1122,57336
124	unspecific	QARMVSKSK	N-Term(Gln->pyro-Glu); K7(Thio-)	1105,54891
124	unspecific	QARMVSKSK	N-Term(Gln->pyro-Glu); K7(Thio-); K9(Thio-)	1193,54651
124	unspecific	QARMVSKSK	M4(Oxidation); K7(Thio-); K9(Thio-)	1226,56879
124	unspecific	QARMVSKSKDGTGS	K7(Thio-); K9(Thio-)	1627,72435
127	unspecific	MVSKSKDGTGS	K4(Thio-); K6(Thio-)	1272,52648
147	unspecific	GKTKIATPR	K2(Thio-); K4(Thio-)	1147,59564
157	unspecific	AAPPGQKGQANATR	K7(Thio-)	1454,71737
159	unspecific	PPGQKGQANATR	K5(Thio-)	1312,64092
172	unspecific	PAKTPPAPK	K3(Thio-)	994,538865
174	unspecific	KTPPAPKTPPSSGEPPK	N-Term(Thio-); K7(Thio-)	1891,92823
174	unspecific	KTPPAPKTPPSSGEPPKSG	K1(Thio-)	1947,98626
224	unspecific	KKVAVVR	N-Term(Thio-); K2(Thio-)	975,547708
229	unspecific	VRTPPKSPSSAKSRLQTAPVPMP	K6(Thio-); K12(Thio-); M22(Oxidation)	2624,33967
232	unspecific	PPKSPSSAKSR	K3(Thio-); K9(Thio-)	1317,62703
238	unspecific	SAKSRLQTAPVPMP	N-Term(Thio-)	1570,80723
238	unspecific	SAKSRLQTAPVPMP	K3(Thio-); M13(Oxidation)	1586,80062
240	unspecific	KSRLQTAPVPMP	N-Term(Thio-)	1412,73739
240	unspecific	KSRLQTAPVPMP	N-Term(Thio-); M11(Oxidation)	1428,73623
253	unspecific	PKKVAVVR	K2(Thio-); K3(Thio-)	1072,60014
259	unspecific	KIGSTENLK	K1(Thio-)	1077,55992
261	unspecific	GSTENLKHQPGGGK	K7(Thio-)	1497,71243
280	unspecific	KKLDLSNVQSK	N-Term(Thio-); K2(Thio-)	1435,72837
306	unspecific	VQIVYKPVDLSK	K6(Thio-)	1476,81205
306	unspecific	VQIVYKPV		945,577304
308	unspecific	IVYKPVDLSK	N-Term(Thio-); K4(Thio-)	1337,69232
308	unspecific	IVYKPVDLSK	K4(Thio-)	1249,68727
309	unspecific	VYKPVDLSK	K3(Thio-)	1136,60348
311	unspecific	KPVDLSK	N-Term(Thio-); K7(Thio-)	962,469585
311	unspecific	KPVDLSKVTSK	K1(Thio-); K7(Thio-)	1377,71123
311	unspecific	KPVDLSKVTSK	K7(Thio-)	1289,7134
314	AspN + unspecific	DLSKVTSK	K4(Thio-)	965,497619
331	unspecific	KPGGGQVEVK	N-Term(Thio-)	1086,56173
369	unspecific	KKIETHK	K1(Thio-); K2(Thio-)	1059,53111
391	unspecific	EIVYKSPVVSG	K5(Thio-)	1265,64539
391	unspecific	EIVYKSPVVSG		1177,6435
395	unspecific	KSPVVSGDTSPR	N-Term(Thio-)	1317,64553

Unspecific peptides detected at least in one sample are shown, with the corresponding first amino acid residue and N-terminal modifications (numbering of N-terminal residues correspond to the N-terminal cleavage site identified).

Table. S1B

Residue position	N-ter cleavage	Detected peptide	Modification (s)	MH+ Da
13	AspN	DHAGTYGLGDR	N-Term(Thio-)	1249,52514
13	AspN	DHAGTYGLGDRK	K12(Thio-)	1377,61942
22	AspN	DRKDQGGYTMHQ	N-Term(Thio-)	1523,6374
25	AspN	DQGGYTMHQDQEGDTDAGLK		2165,90082
34	AspN	DQEGDTDAGLKESPLQTPTE	K11(Thio-)	2218,96318
38	AspN	DTDAGLKESPLQTPTE	K7(Thio-)	1789,81828
139	AspN	DKKAKGA	K2(Thio-); K3(Thio-); K5(Thio-)	981,420266
193	AspN	DRSGYSSPGSPGTPGSR		1664,76377
252	AspN	DLKNVKSK	K6(Thio-); K8(Thio-)	1107,55387
252	AspN	DLKNVKSKI	K6(Thio-)	1132,63972
252	AspN	DLKNVKSKIGSTE	N-Term(Thio-); K6(Thio-); K8(Thio-)	1682,77876
252	AspN	DLKNVKSKIGSTE	K3(Thio-); K8(Thio-)	1594,77824
252	AspN	DLKNVKSKIGSTE	K6(Thio-)	1506,7844
283	AspN	DLSNVQSK	K8(Thio-)	978,456404
283	AspN	DLSNVQSKC	K8(Thio-); C9(Carbamidomethyl)	1138,48566
283	AspN	DLSNVQSKC		993,465083
283	AspN	DLSNVQSKCG	K8(Thio-)	1138,48566
283	AspN	DLSNVQSKCGSK	K12(Thio-)	1353,61378
283	AspN	DLSNVQSKCGSK	K8(Thio-); K12(Thio-)	1441,61087
283	AspN	DLSNVQSKCGSK		1265,61538
295	AspN	DNIKHVPGGGSV	N-Term(Thio-)	1267,61057
295	AspN	DNIKHVPGGGSVQ	K4(Thio-)	1395,66632
295	AspN	DNIKHVPGGGSVQI	K4(Thio-)	1508,75308
295	AspN	DNIKHVPGGGSVQIV	K4(Thio-)	1607,8191
295	AspN	DNIKHVPGGGSVQIVYKPV	N-Term(Thio-); K17(Thio-)	2183,09418
295	AspN	DNITHVPGGGNK	K12(Thio-)	1296,6025
295	AspN	DNITHVPGGGNKK	K12(Thio-); K13(Thio-)	1512,69222
295	AspN	DNITHVPGGGNKK	K12(Thio-)	1424,69464
295	AspN	DNITHVPGGGNKKI	K12(Thio-); K13(Thio-)	1625,77583
295	AspN	DNITHVPGGGNKKIE	K12(Thio-)	1666,81992
295	AspN	DNITHVPGGGNKKIET	K12(Thio-); K13(Thio-)	1855,86555
295	AspN	DNITHVPGGGNKKIETH	K12(Thio-); K13(Thio-)	1992,92367
295	AspN	DNITHVPGGGNKKIETH	K12(Thio-)	1904,92351
295	AspN	DNITHVPGGGNKKIETHK	K12(Thio-); K13(Thio-); K18(Thio-)	2209,0186
295	AspN	DNITHVPGGGNKKIETHK	K12(Thio-); K13(Thio-)	2121,02158
295	AspN	DNITHVPGGGNKKIETHKL	K12(Thio-); K13(Thio-); K18(Thio-)	2322,09952
295	AspN	DNITHVPGGGNKKIETHKLTFR	K12(Thio-); K13(Thio-); K18(Thio-)	2726,31926
314	AspN	DLSKVTSK	K4(Thio-); K8(Thio-)	1053,49435
314	AspN	DLSKVTSKC	N-Term(Thio-); K8(Thio-)	1156,50549
314	AspN	DLSKVTSKC	K4(Thio-); K8(Thio-); C9(Carbamidomethyl)	1213,52594
314	AspN	DLSKVTSKC	K8(Thio-); C9(Carbamidomethyl)	1125,52773
314	AspN	DLSKVTSKCG	K4(Thio-); K8(Thio-)	1213,52651
314	AspN	DLSKVTSKCG	K4(Thio-)	1125,5277
314	AspN	DLSKVTSKCGSL	K4(Thio-); K8(Thio-); C9(Carbamidomethyl)	1470,66377
314	AspN	DLSKVTSKCGSL	C9(Carbamidomethyl)	1294,66724
314	AspN	DLSKVTSKCGSL		1237,64536
314	AspN	DLSKVTSKCGSLG	N-Term(Thio-); K8(Thio-)	1470,66269
314	AspN	DLSKVTSKCGSLG	K8(Thio-)	1382,66476
314	AspN	DLSKVTSKCGSLG		1294,66741
348	AspN	DRVQSKIG	K6(Thio-)	990,503636
348	AspN	DRVQSKIGS	K6(Thio-)	1077,53519
348	AspN	DRVQSKIGSL	K6(Thio-)	1190,61933
348	AspN	DRVQSKIGSL		1102,62239
348	AspN	DRVQSKIGSLD	K6(Thio-)	1305,6461
387	AspN	DHGAEIVYKSPVVSG	K9(Thio-)	1645,78744
387	AspN	DHGAEIVYKSPVVSG		1557,78865
402	AspN	DTSPRHLSNVSSTGSI	N-Term(Thio-)	1745,81177
402	AspN	DTSPRHLSNVSSTGSI		1657,81427
402	AspN	DTSPRHLSNVSSTGSIDMV		2002,94662

Asp-N peptides detected in at least one sample are shown, with the corresponding first amino acid residue and N-terminal modifications (numbering of N-terminal residues correspond to the N-terminal cleavage site identified).

Table. S1C

Residue position	N-ter cleavage	Detected peptide	Modification (s)	MH+ Da
6	trypsin	QEFEVMEDHAGTYGLGDR	N-Term(Gln->pyro-Glu); M6(Oxidation)	2052,85937
6	trypsin	QEFEVMEDHAGTYGLGDR		2053,89274
6	trypsin	QEFEVMEDHAGTYGLGDR	M6(Oxidation)	2069,88523
24	trypsin	KDQGGYTMH	K1(Thio-)	1124,44931
24	trypsin	KDQGGYTMHQDQEGDTDAGLK	K1(Thio-)	2381,99814
24	trypsin	KDQGGYTMHQDQEGDTDAGLK	K1(Thio-); M8(Oxidation)	2397,99096
68	trypsin	STPTAEDVTAPLVDEGAPGK		1954,96381
127	trypsin	MVSKSKDGTGSDDK	K4(Thio-); K6(Thio-)	1630,67175
127	trypsin	MVSKSKDGTGSDDK	M1(Oxidation); K4(Thio-); K6(Thio-)	1646,66567
131	trypsin	SKDGTGSDDKK	N-Term(Thio-); K11(Thio-)	1313,53457
142	trypsin	AKGADGKTK	N-Term(Thio-); K7(Thio-)	1051,49166
144	trypsin	GADGKTKIATPR	K5(Thio-); K7(Thio-)	1390,67739
144	trypsin	GADGKTKIATPR	N-Term(Thio-); K5(Thio-); K7(Thio-)	1478,6793
156	trypsin	GAAPPGQKGQ	K8(Thio-)	998,472517
156	trypsin	GAAPPGQKGQAN	K8(Thio-)	1183,55277
156	trypsin	GAAPPGQKGQANATR	K8(Thio-)	1511,73566
171	trypsin	IPAKTPPAPK	K4(Thio-)	1107,62297
171	trypsin	IPAKTPPAPKTPPSSGEPPK	K4(Thio-); K10(Thio-)	2173,10375
175	trypsin	TPPAPKTPPSSGEPPK	K6(Thio-)	1675,83398
175	trypsin	TPPAPKTPPSSGEPPKSGDR	K6(Thio-); K16(Thio-)	2179,01493
175	trypsin	TPPKSPSSAK	K4(Thio-)	1087,54494
175	trypsin	TPPKSPSSAK	N-Term(Thio-); K10(Thio-)	1175,54235
175	trypsin	TPPKSPSSAKSR	K4(Thio-); K10(Thio-)	1418,6765
175	trypsin	TPPSSGEPPKSGDR	K10(Thio-)	1499,67886
195	trypsin	SGYSSPGSPGTPGSR		1393,63351
195	trypsin	SGYSSPGSPGTPGSR	N-Term(Thio-)	1481,63153
212	trypsin	TPSLPTPPTR		1066,58939
212	trypsin	TPSLPTPPTREPK		1420,77922
222	trypsin	EPKKVAVVR	N-Term(Glu->pyro-Glu); K4(Thio-)	1095,63297
222	trypsin	EPKKVAVVR	N-Term(Thio-); K4(Thio-)	1201,64156
222	trypsin	EPKKVAVVR	N-Term(Glu->pyro-Glu); K3(Thio-); K4(Thio-)	1183,63204
241	trypsin	SRLQTAPVPMP		1196,64678
243	trypsin	LQTAPVPMP		953,511077
243	trypsin	LQTAPVPMPDLK		1309,71765
243	trypsin	LQTAPVPMPDLK	M8(Oxidation)	1325,71345
243	trypsin	LQTAPVPMPDLK	K12(Thio-)	1397,71714
243	trypsin	LQTAPVPMPDLKN	K12(Thio-)	1511,75992
243	trypsin	LQTAPVPMPDLKN	M8(Oxidation); K12(Thio-)	1527,75656
243	trypsin	LQTAPVPMPDLKNVK	K15(Thio-)	1738,92395
243	trypsin	LQTAPVPMPDLKNVK	M8(Oxidation); K12(Thio-)	1754,91702
243	trypsin	LQTAPVPMPDLKNVKS	M8(Oxidation); K12(Thio-); K15(Thio-)	1929,94724
243	trypsin	LQTAPVPMPDLKNVKSK	M8(Oxidation); K12(Thio-); K17(Thio-)	2058,0458
255	trypsin	NVKSKIGSTENLK	K3(Thio-); K5(Thio-)	1593,79727
258	trypsin	SKIGSTENLK	K2(Thio-)	1164,59137
258	trypsin	SKIGSTENLK	N-Term(Acetyl); K2(Thio-)	1206,61486
258	trypsin	SKIGSTENLKHQPGGGK	N-Term(Thio-); K10(Thio-)	1913,9199
260	trypsin	IGSTENLKH	K8(Thio-)	1086,52457
260	trypsin	IGSTENLKHQPGGGK	K8(Thio-)	1610,79249
268	trypsin	HQPGGGKVQI	K7(Thio-)	1108,5573
268	trypsin	HQPGGGKVQII	K7(Thio-)	1221,6413
268	trypsin	HQPGGGKVQIIN	N-Term(Thio-)	1335,68333
268	trypsin	HQPGGGKVQIINK	K7(Thio-)	1463,77678
281	trypsin	KLDLSNVQSK	N-Term(Thio-)	1219,63406
282	trypsin	LDLSNVQSK		1003,54199
282	trypsin	LDLSNVQSKCGSK	K9(Thio-)	1466,6971
299	trypsin	HVPGGGSVQIV		1049,57411
299	trypsin	HVPGGGSVQIVY		1212,6364
299	trypsin	HVPGGGSVQIVYKPV		1536,8522
299	trypsin	HVPGGGSVQIVYKPVDLSK		1980,08947
312	trypsin	PVDLSKVTSK	K6(Thio-)	1161,61832
318	trypsin	VTSKCGSL	N-Term(Thio-); K4(Thio-); C5(Carbamidomethyl)	1027,43011
318	trypsin	VTSKCGSLGN	K4(Thio-)	1053,47119
322	trypsin	CGSLGNIHHKPGGGQVEVK		1916,97431
322	trypsin	CGSLGNIHHKPGGGQVEVK	K10(Thio-)	2004,97098
328	trypsin	IHHKPGGGQVEVK	K4(Thio-)	1473,76243
332	trypsin	PGGGQVEVKSEK	K9(Thio-)	1302,6343
341	trypsin	SEKLDFK	K3(Thio-)	954,460063
341	trypsin	SEKLDFKDR	K3(Thio-); K7(Thio-)	1313,58737
350	trypsin	VQSKIGSLDN	K4(Thio-)	1148,56166
350	trypsin	VQSKIGSLDNIT	K4(Thio-)	1362,69425
350	trypsin	VQSKIGSLDNITH	K4(Thio-)	1499,75082
350	trypsin	VQSKIGSLDNITHVPGGGN	N-Term(Thio-)	1980,98008
350	trypsin	VQSKIGSLDNITHVPGGGNK	K4(Thio-)	2109,07556
350	trypsin	VQSKIGSLDNITHVPGGGNKK	K4(Thio-); K20(Thio-)	2325,16676
354	trypsin	IGSLDNITHVPGGGNK		1578,82346
354	trypsin	IGSLDNITHVPGGGNKK	K17(Thio-)	1794,91563
371	trypsin	IETHKLTFR	K5(Thio-)	1232,6452
380	trypsin	ENAKAKTDHGAEIVY	N-Term(Thio-); K4(Thio-)	1821,81108
380	trypsin	ENAKAKTDHGAEIVYK	K4(Thio-); K6(Thio-)	1949,90891
384	trypsin	AKTDHGAEI	K2(Thio-)	1029,46803
384	trypsin	AKTDHGAEIV	K2(Thio-)	1128,53499
384	trypsin	AKTDHGAEIVY	N-Term(Thio-)	1291,59716
384	trypsin	AKTDHGAEIVYK	N-Term(Thio-)	1419,69386
386	trypsin	TDHGAEIVYK	N-Term(Thio-)	1220,56102
386	trypsin	TDHGAEIVYKSPVVSGDTSPR	K10(Thio-)	2303,09623
407	trypsin	HLSNVSSTGSIDMVDSPQLA		2057,98125

Tryptic peptides detected in at least one sample are shown, with the corresponding first amino acid residue and N-terminal modifications (numbering of N-terminal residues correspond to the N-terminal cleavage site identified).

Table. S2

Summary of antibodies used

Antibody	Species	Dilution	Application(s)	Supplier
Tau-Nter (total Tau, 1-19)	Rabbit	1/10 000	WB	Homemade
Tau-Cter (total Tau, 426-441)	Rabbit	1/10 000 – 1/1000	WB - ICC	Homemade
Tau-5	Mouse	1/1000 - 1/100	WB - IP	Invitrogen
PSer396	Rabbit	1/10 000	WB	Invitrogen
AT180	Mouse	1/500	WB	Pierce
12E8	Rabbit	1/1000	WB	Homemade
α-tubulin (Total-tubulin)	Mouse	1/1000 - 1/200	WB - ICC	Sigma
Acetyl-α-tubulin	Mouse	1/2000 - 1/200	WB - ICC	Sigma
Tyrosinated-α-tubulin	Mouse	1/1000	WB	Sigma
Detyrosinated -α-tubulin	Mouse	1/2000	WB	Abcam
ß-actin	Mouse	1/10 000	WB	Sigma
GAPDH	Rabbit	1/10 000	WB	Santa Cruz
Lamin-B	Goat	1/1000	WB	Santa Cruz
HDAC6	Rabbit	1/1000	WB	Cell signaling

Fig. S1

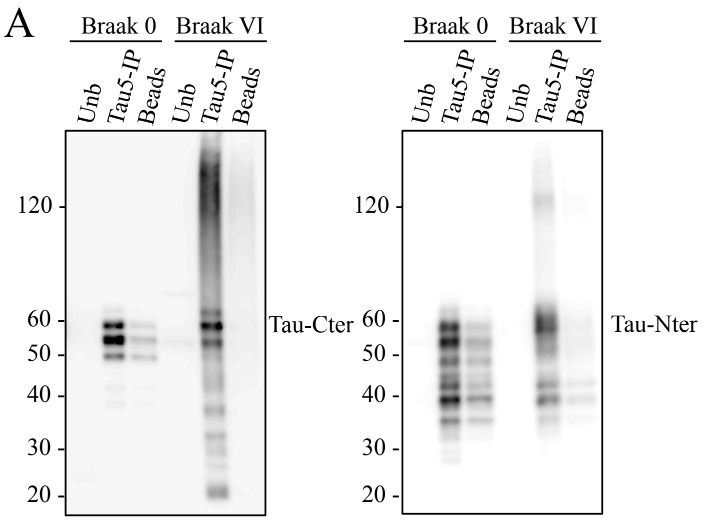
A
Braak 0
Braak VI
Unb
Tau5-IP
Beads
Unb
Tau5-IP
Beads
120
60
50
40
30
20
Tau-Cter
Braak 0
Braak VI
Unb
Tau5-IP
Beads
Unb
Tau5-IP
Beads
120
60
50
40
30
20
Tau-Nter

Fig. S2

A

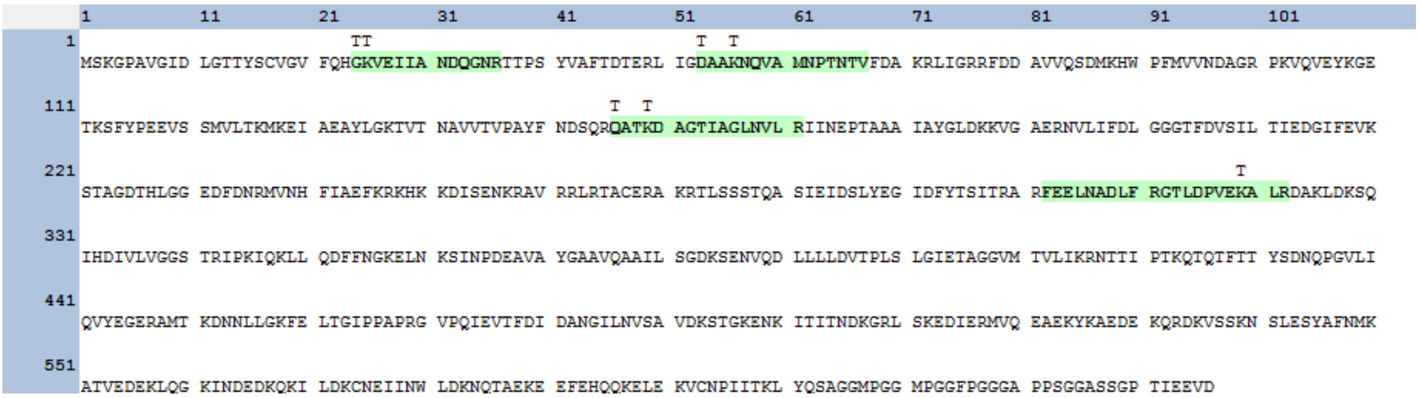
1 11 21 31 41 51 61 71 81 91 101
1
TT T T
MSKGPAVGID LGTTYSCVGV FQHGKVEIIA NDQGNRTTPS YVAFTDTERL IGDAAKNQVA MNPTNTVFDA KRLIGRRFDD AVVQSDMKHW PFMVVNDAGR PKVQVEYKGE
111
T T
TKSFYPEEVS SMVLTKMKEI AEAYLGKTVT NAVVTVPAYF NDSQRQATKD AGTIAGLNVL RIINEPTAAA IAYGLDKKVG AERNVLIFDL GGGTFDVSIL TIEDGIFEVK
221
T
STAGDTHLGG EDFDNRMVNH FIAEFKRKHK KDISENKRAV RRLRTACERA KRTLSSSTQA SIEIDSLYEG IDFYTSITRA RFEELNADLF RGTLDPVEKA LRDAKLDKSQ
331
IHDIVLVGGS TRIPKIQKLL QDFFNGKELN KSINPDEAVA YGAAVQAAIL SGDKSENVQD LLLLDVTPLS LGIETAGGVM TVLIKRNTTI PTKQTQTFTT YSDNQPGVLI
441
QVYEGERAMT KDNNLLGKFE LTGIPPAPRG VPQIEVTFDI DANGILNVSA VDKSTGKENK ITITNDKGRL SKEDIERMVQ EAEKYKAEDE KQRDKVSSKN SLESYAFNMK
551
ATVEDEKLQG KINDEDKQKI LDKCNEIINW LDKNQTAEKE EFEHQQKELE KVCNPIITKL YQSAGGMPGG MPGGFPGGGA PPSGGASSGP TIEEVD

B

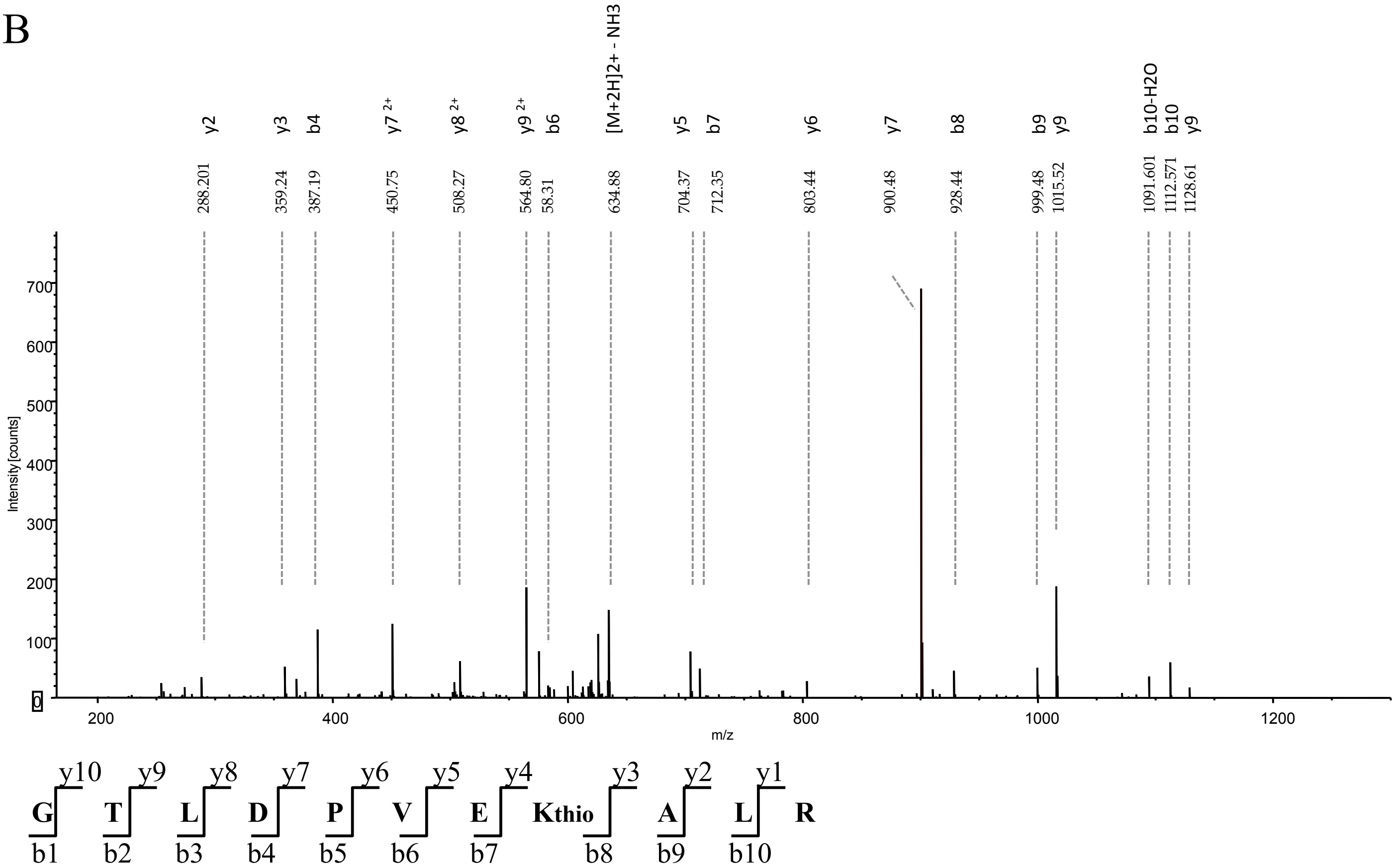
y2
y3
b4
y7 2+
y8 2+
y9 2+
b6
[M+2H]2+ - NH3
y5
b7
y6
y7
b8
b9
y9
b10-H2O
b10
y9
288.201
359.24
387.19
450.75
508.27
564.80
58.31
634.88
704.37
712.35
803.44
900.48
928.44
999.48
1015.52
1091.601
1112.571
1128.61
Intensity [counts]
700
600
500
400
300
200
100
0
200
400
600
800
1000
1200
m/z
y10 y9 y8 y7 y6 y5 y4 y3 y2 y1
G T L D P V E Kthio A L R
b1 b2 b3 b4 b5 b6 b7 b8 b9 b10

Fig. S3

A

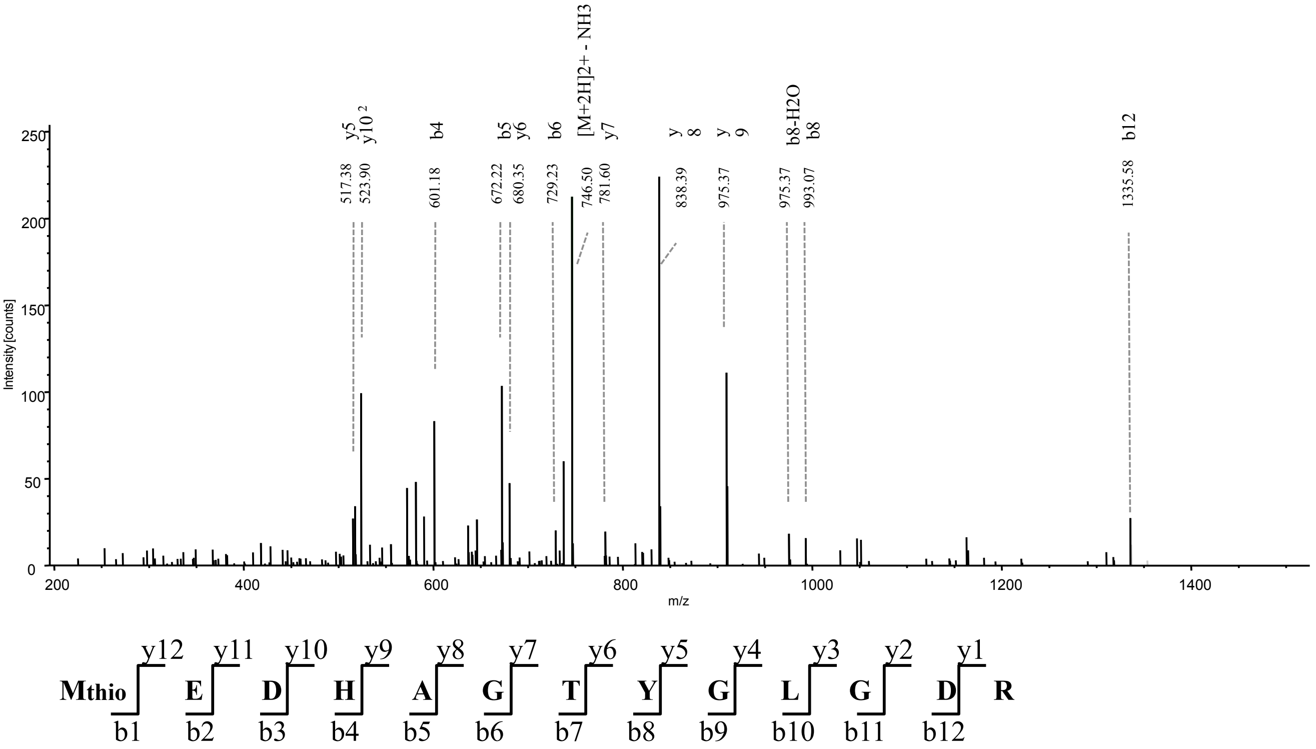
y5
y10 2
b4
b5
y6
b6
[M+2H]2+ - NH3
y7
y 8
y 9
b8-H2O
b8
b12
517.38
523.90
601.18
672.22
680.35
729.23
746.50
781.60
838.39
975.37
975.37
993.07
1335.58
Intensity [counts]
m/z
Mthio E D H A G T Y G L G D R
y12 y11 y10 y9 y8 y7 y6 y5 y4 y3 y2 y1
b1 b2 b3 b4 b5 b6 b7 b8 b9 b10 b11 b12

B

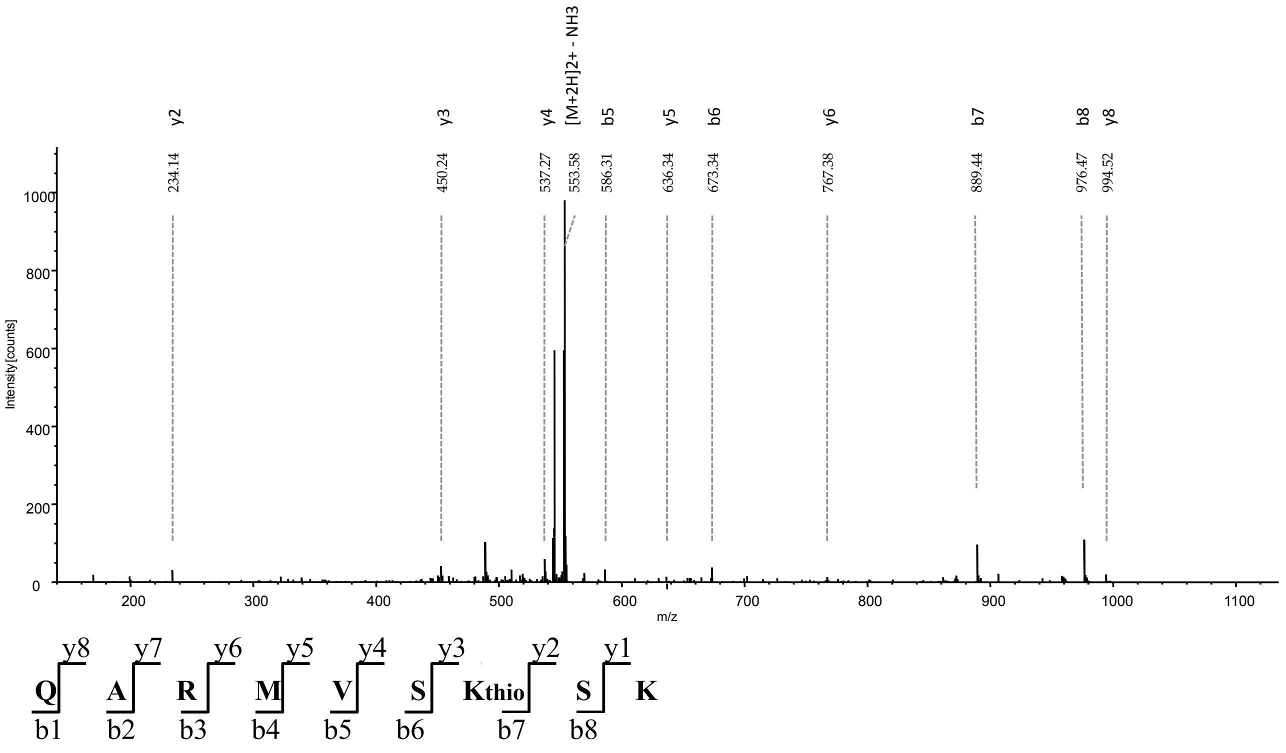
y2
y3
y4
[M+2H]2+ - NH3
b5
y5
b6
y6
b7
b8
y8
234.14
450.24
537.27
553.58
586.31
636.34
673.34
767.38
889.44
976.47
994.52
Intensity [counts]
m/z
Q A R M V S Kthio S K
y8 y7 y6 y5 y4 y3 y2 y1
b1 b2 b3 b4 b5 b6 b7 b8

Fig. S4

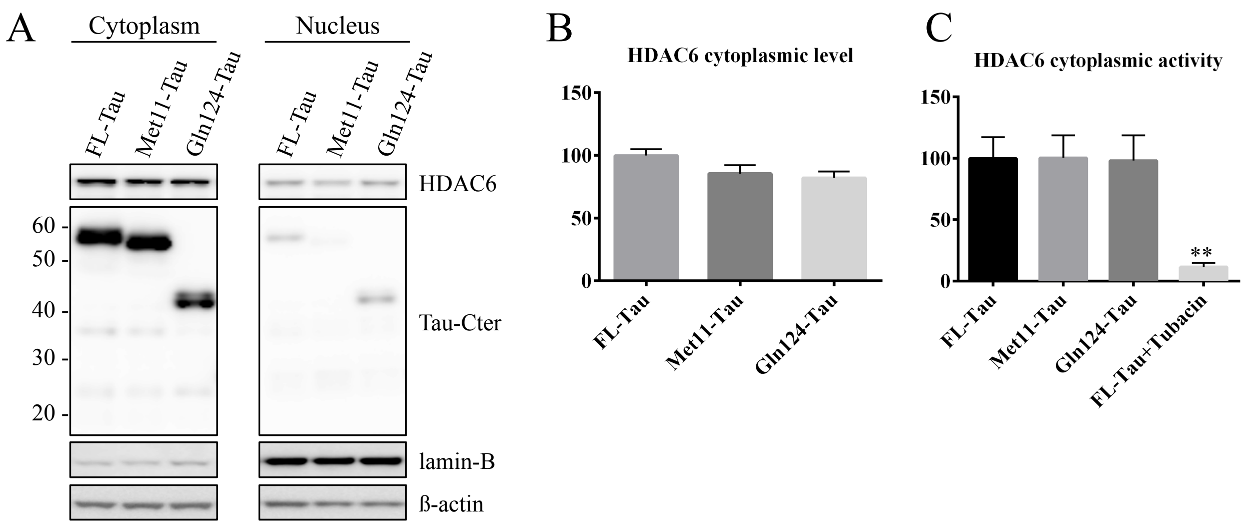
A
Cytoplasm
Nucleus
FL-Tau
Met11-Tau
Gln124-Tau
HDAC6
60
50
40
30
20
Tau-Cter
lamin-B
ß-actin
B
HDAC6 cytoplasmic level
150
100
50
0
FL-Tau
Met11-Tau
Gln124-Tau
C
HDAC6 cytoplasmic activity
150
100
50
0
**
FL-Tau
Met11-Tau
Gln124-Tau
FL-Tau+Tubacin

Fig. S5

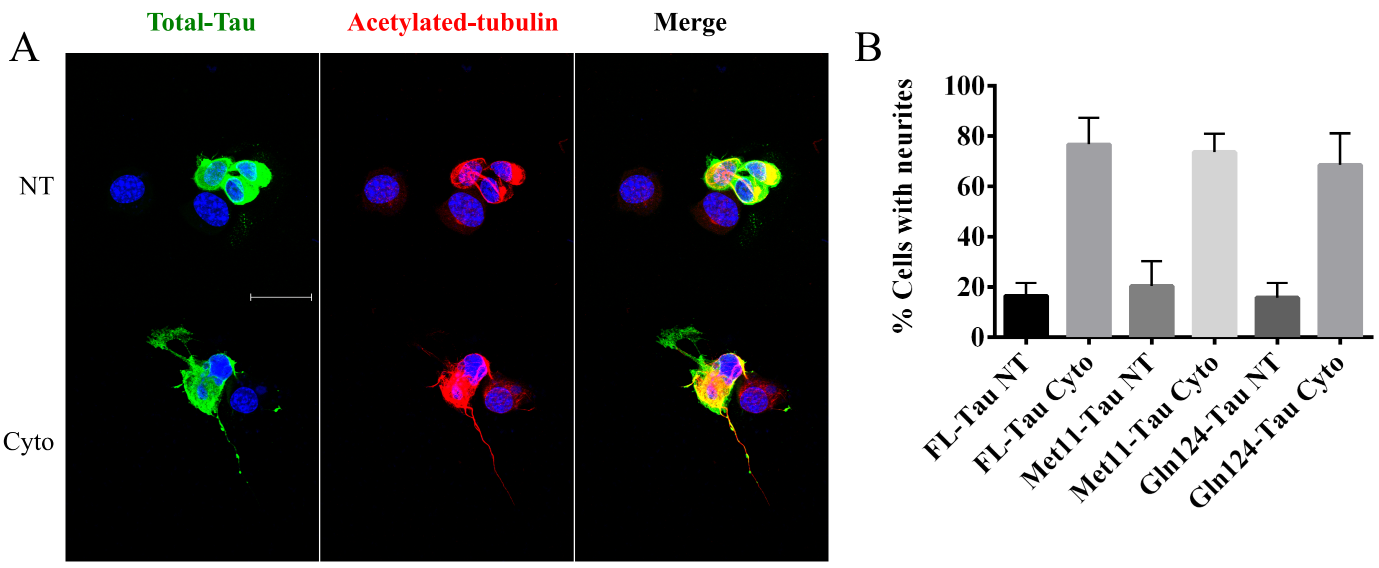

A
Total-Tau
Acetylated-tubulin
Merge
NT
Cyto
B
% Cells with neurites
100
80
60
40
20
0
FL-Tau NT
FL-Tau Cyto
Met11-Tau NT
Met11-Tau Cyto
Gln124-Tau NT
Gln124-Tau Cyto

Fig. S6

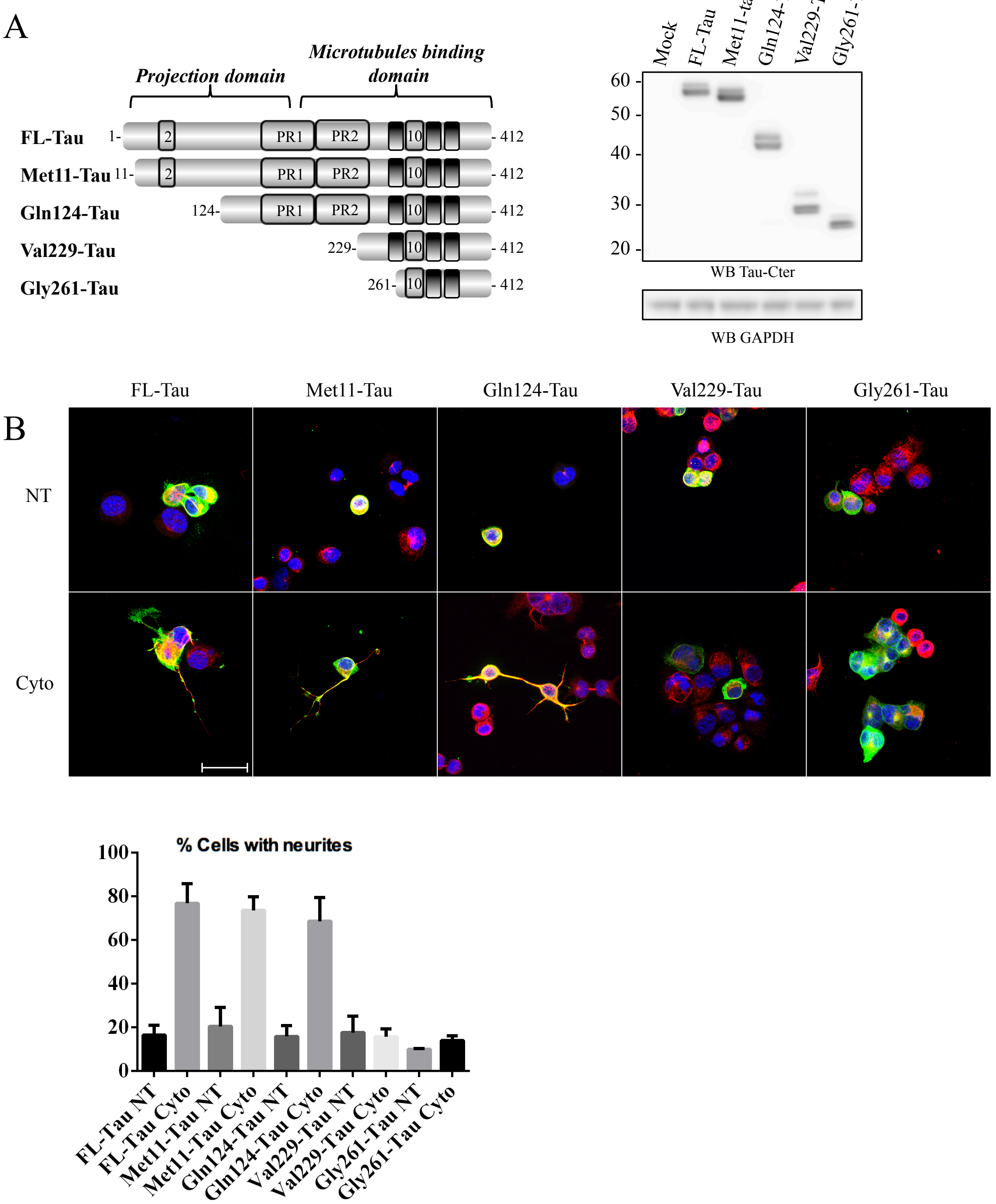

A
Projection domain
Microtubules binding domain
FL-Tau
Met11-Tau
Gln124-Tau
Val229-Tau
Gly261-Tau
1-
11-
124-
229-
261-
- 412
2
PR1
PR2
10
Mock
FL-Tau
Met11-tau
Gln124-Tau
Val229-Tau
Gly261-Tau
60
50
40
30
20
WB Tau-Cter
WB GAPDH
B
FL-Tau
Met11-Tau
Gln124-Tau
Val229-Tau
Gly261-Tau
NT
Cyto
% Cells with neurites
100
80
60
40
20
0
FL-Tau NT
FL-Tau Cyto
Met11-Tau NT
Met11-Tau Cyto
Gln124-Tau NT
Gln124-Tau Cyto
Val229-Tau NT
Val229-Tau Cyto
Gly261-Tau NT
Gly261-Tau Cyto

Fig. S6

C

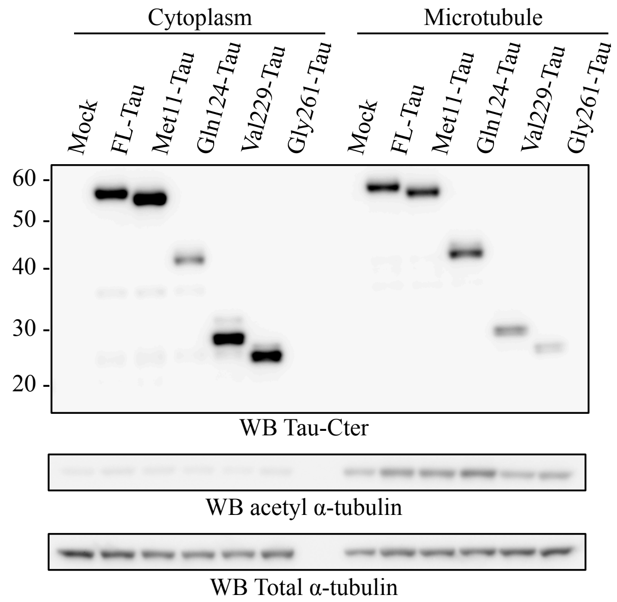

Cytoplasm
Microtubule
Mock
FL-Tau
Met11-Tau
Gln124-Tau
Val229-Tau
Gly261-Tau
Mock
FL-Tau
Met11-Tau
Gln124-Tau
Val229-Tau
Gly261-Tau
60
50
40
30
20
WB Tau-Cter
WB acetyl α-tubulin
WB Total α-tubulin

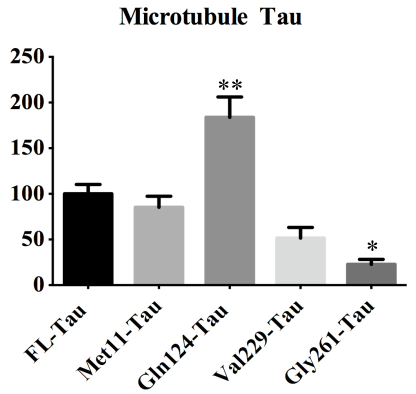

Microtubule Tau
250
200
150
100
50
0
**
*
FL-Tau
Met11-Tau
Gln124-Tau
Val229-Tau
Gly261-Tau

Fig. S7A

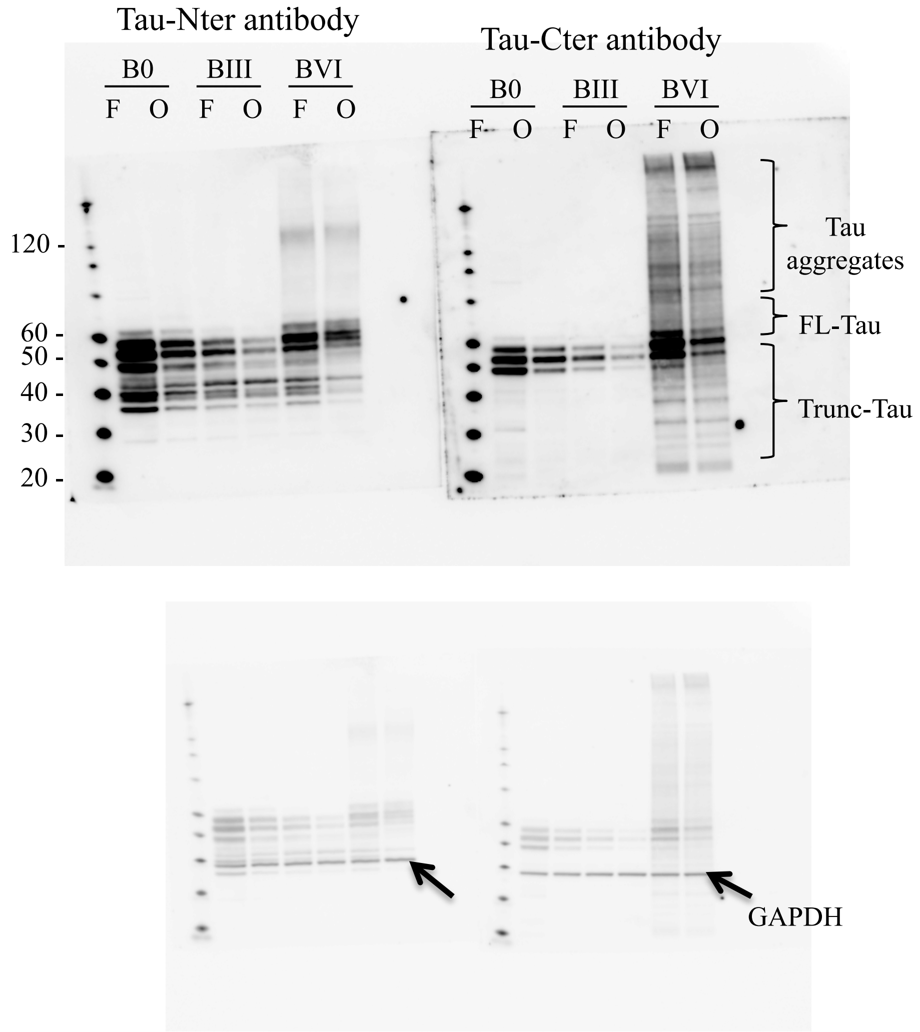

Fig. S7B

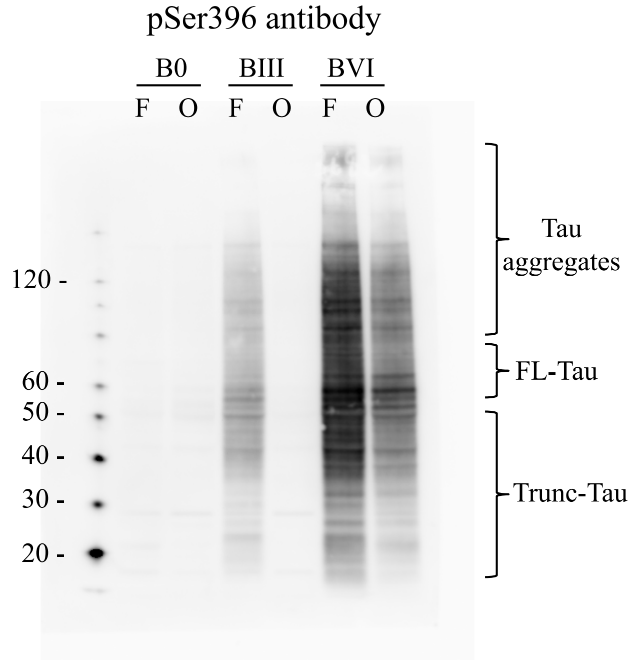

Fig. S8A

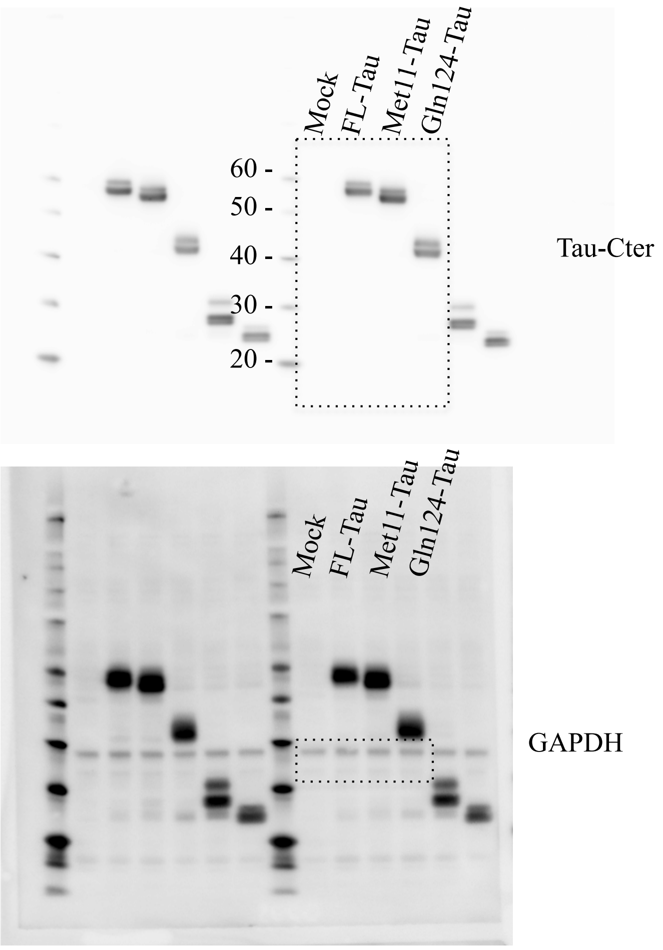
Mock
FL-Tau
Met11-Tau
Gln124-Tau
60 -
50 -
40 -
30 -
20 -
Tau-Cter
Mock
FL-Tau
Met11-Tau
Gln124-Tau
GAPDH

Fig. S8B

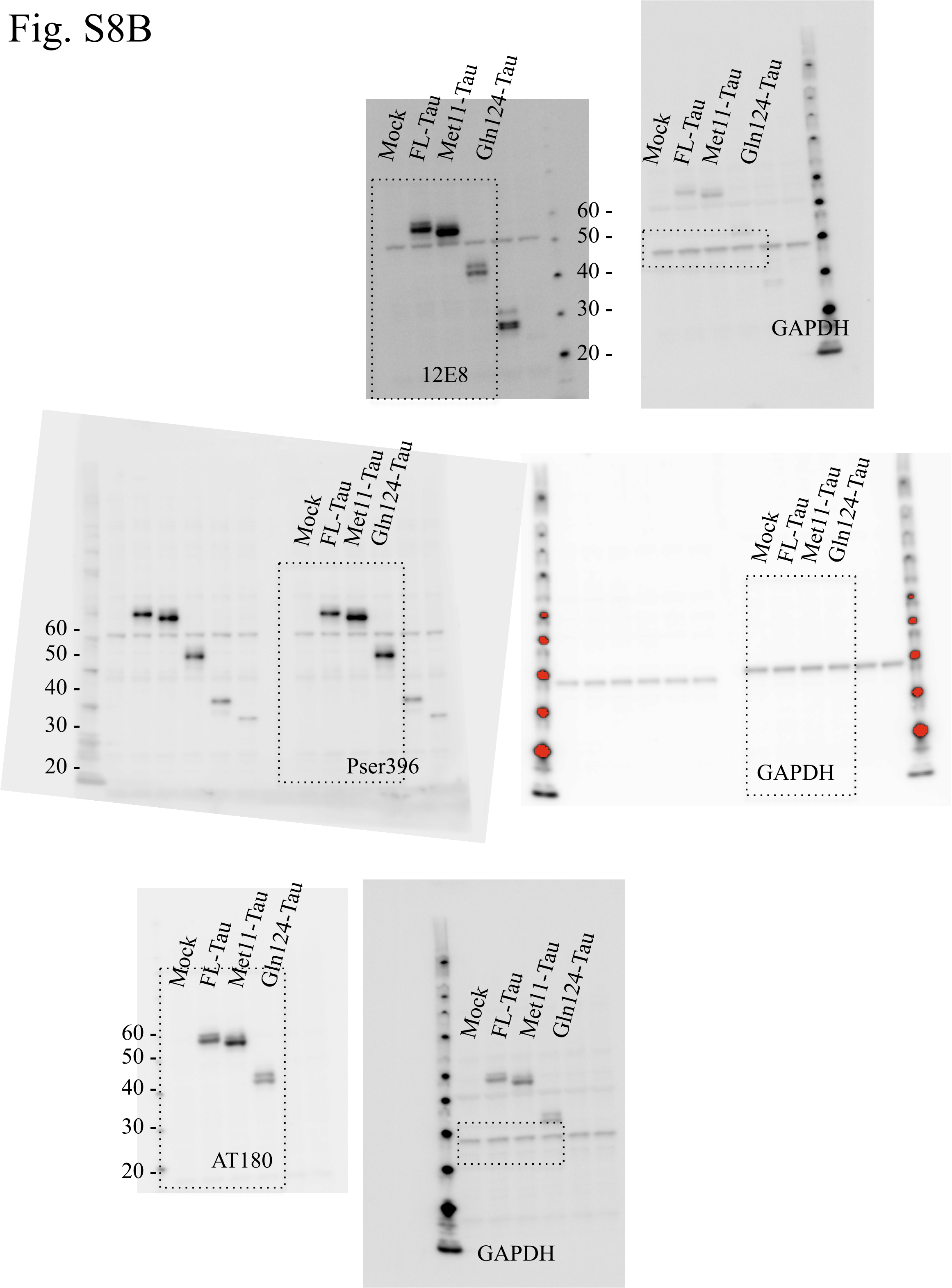
Mock
FL-Tau
Met11-Tau
Gln124-Tau
60 -
50 -
40 -
30 -
20 -
12E8
GAPDH
Pser396
AT180

Fig. S9

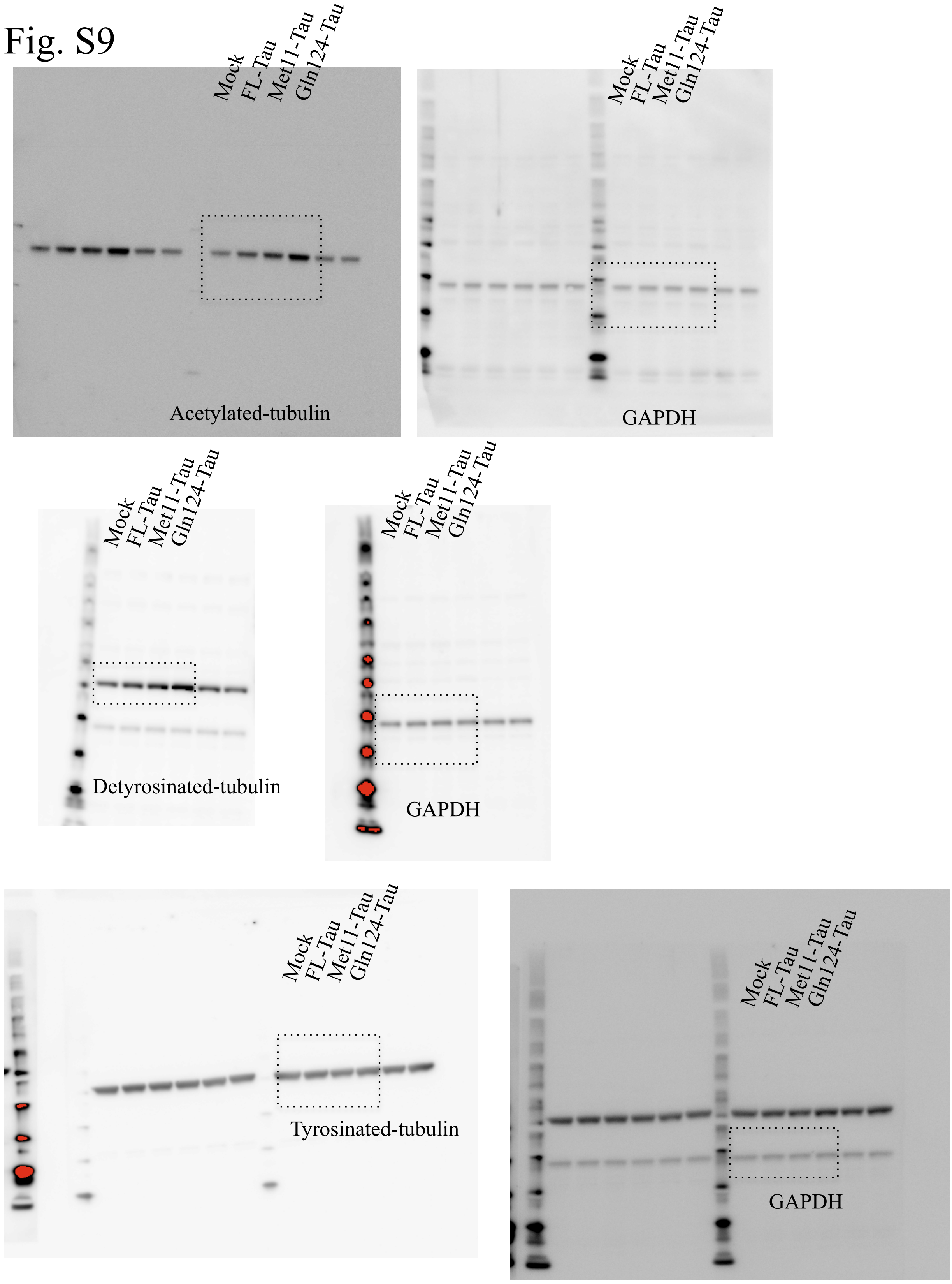
Mock
FL-Tau
Met11-Tau
Gln124-Tau
Acetylated-tubulin
Mock
FL-Tau
Met11-Tau
Gln124-Tau
GAPDH
Mock
FL-Tau
Met11-Tau
Gln124-Tau
Detyrosinated-tubulin
Mock
FL-Tau
Met11-Tau
Gln124-Tau
GAPDH
Mock
FL-Tau
Met11-Tau
Gln124-Tau
Tyrosinated-tubulin
Mock
FL-Tau
Met11-Tau
Gln124-Tau
GAPDH

Fig. S10

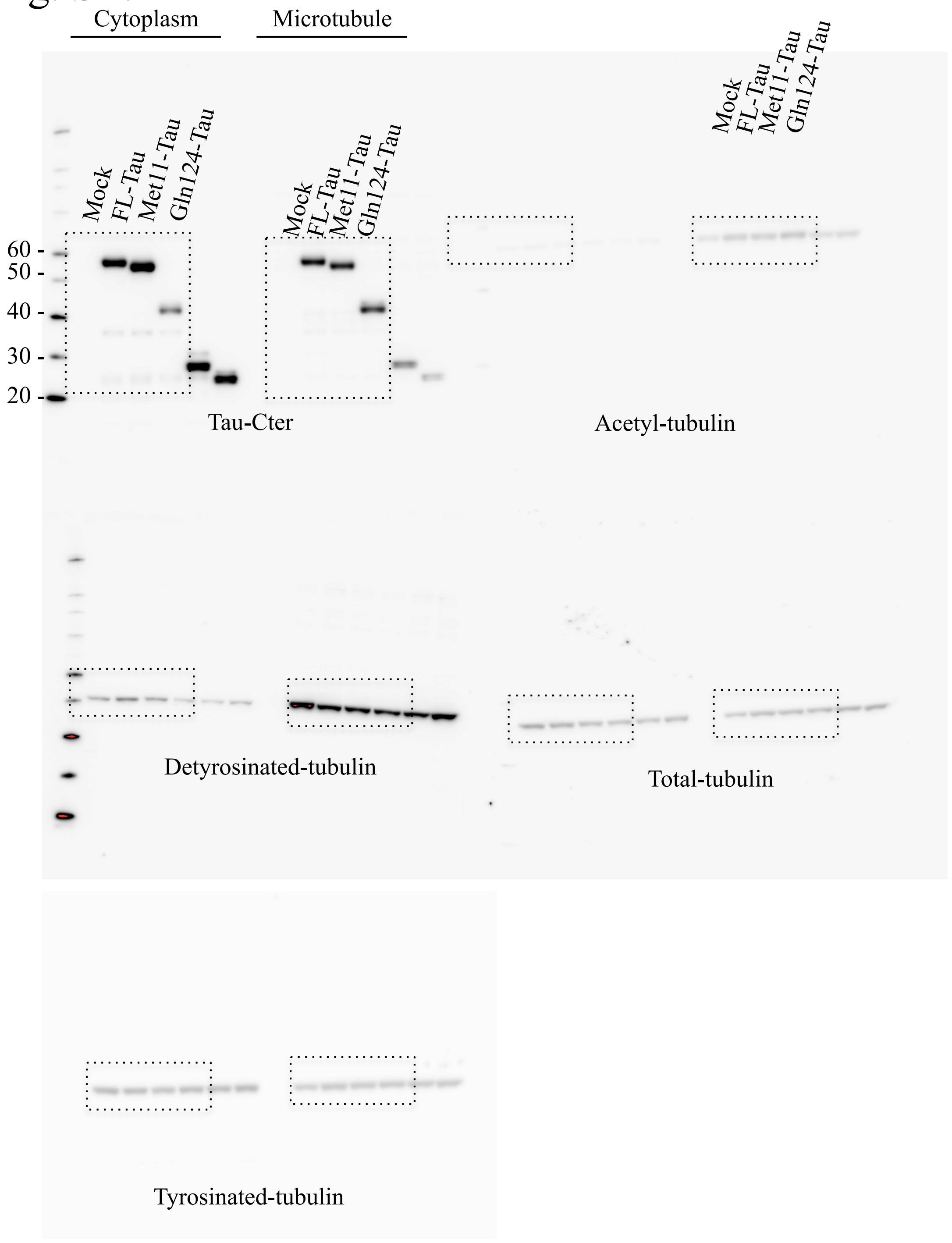Cytoplasm
Microtubule
Mock
FL-Tau
Met11-Tau
Gln124-Tau
Mock
FL-Tau
Met11-Tau
Gln124-Tau
Mock
FL-Tau
Met11-Tau
Gln124-Tau
60
50
40
30
20
Tau-Cter
Acetyl-tubulin
Detyrosinated-tubulin
Total-tubulin
Tyrosinated-tubulin

Fig. S11

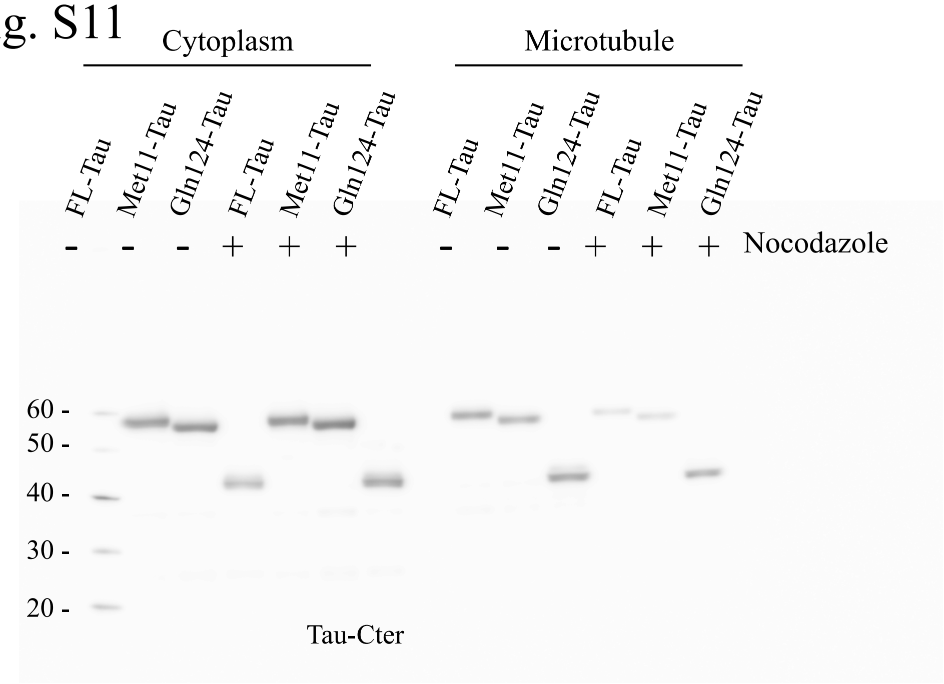
Cytoplasm
Microtubule
FL-Tau
Met11-Tau
Gln124-Tau
FL-Tau
Met11-Tau
Gln124-Tau
FL-Tau
Met11-Tau
Gln124-Tau
FL-Tau
Met11-Tau
Gln124-Tau
- - - + + + - - - + + + Nocodazole
60 -
50 -
40 -
30 -
20 -
Tau-Cter

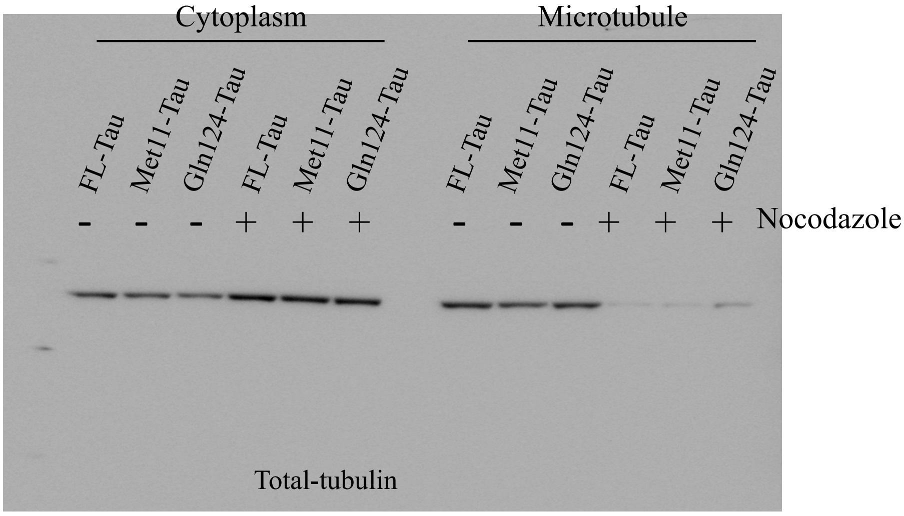
Cytoplasm
Microtubule
FL-Tau
Met11-Tau
Gln124-Tau
FL-Tau
Met11-Tau
Gln124-Tau
FL-Tau
Met11-Tau
Gln124-Tau
FL-Tau
Met11-Tau
Gln124-Tau
- - - + + + - - - + + + Nocodazole
Total-tubulin

Fig. S12

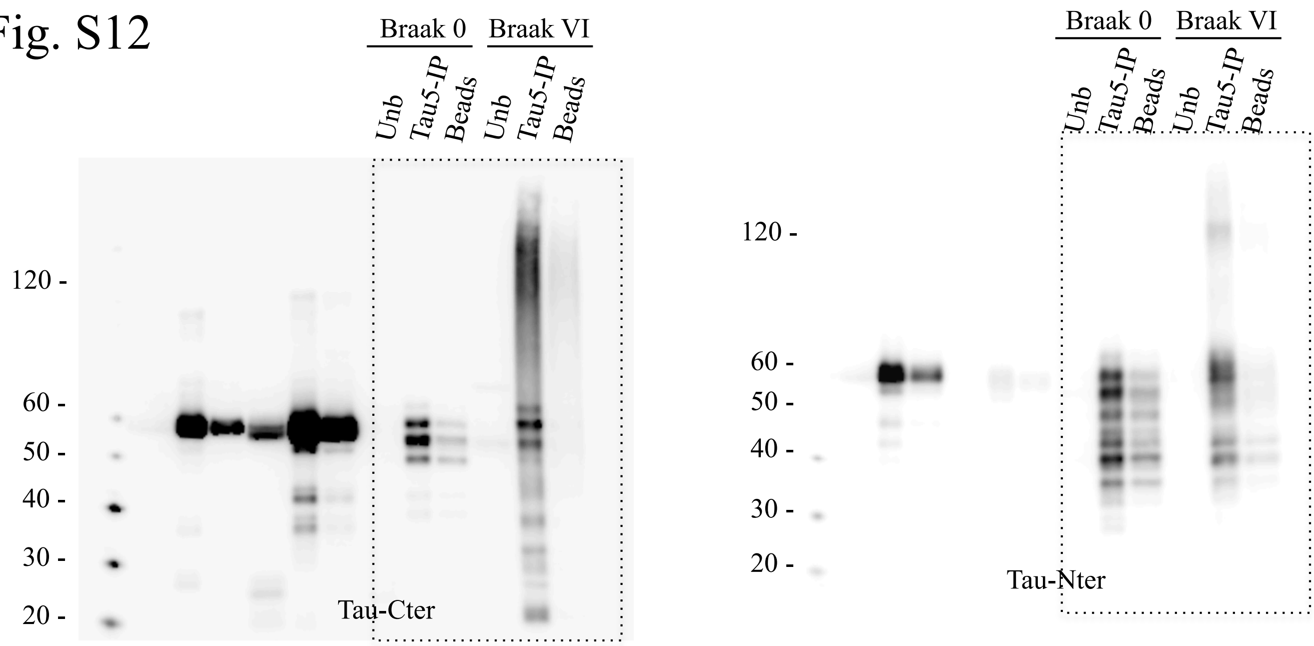
Braak 0
Braak VI
Unb
Tau5-IP
Beads
Unb
Tau5-IP
Beads
120 -
60 -
50 -
40 -
30 -
20 -
Tau-Cter
Braak 0
Braak VI
Unb
Tau5-IP
Beads
Unb
Tau5-IP
Beads
120 -
60 -
50 -
40 -
30 -
20 -
Tau-Nter

Fig. S13

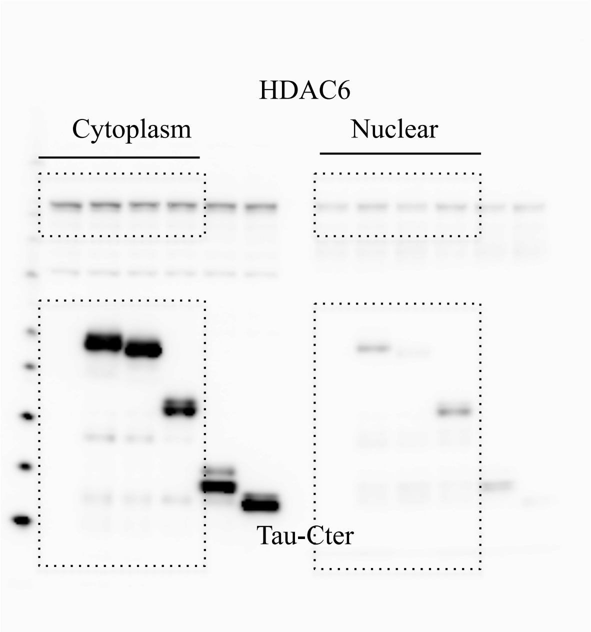
HDAC6
Cytoplasm
Nuclear
Tau-Cter

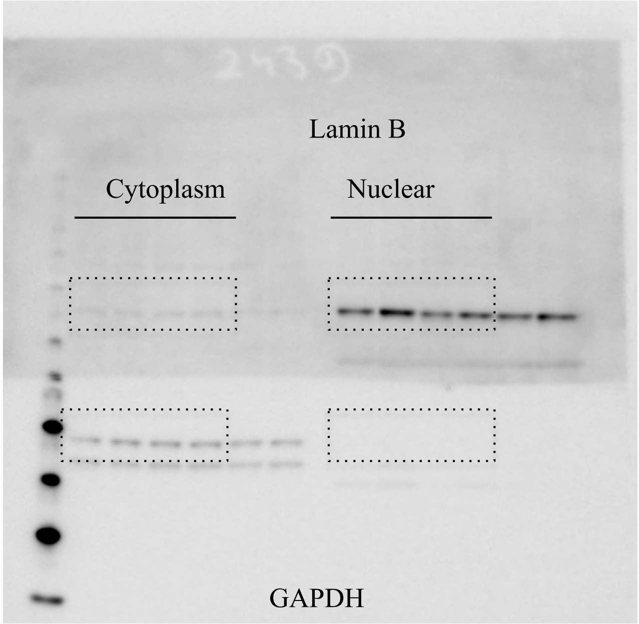
Lamin B
Cytoplasm
Nuclear
GAPDH
